# Characterization of spike glycoprotein of SARS-CoV-2 on virus entry and its immune cross-reactivity with SARS-CoV

**DOI:** 10.1038/s41467-020-15562-9

**Published:** 2020-03-27

**Authors:** Xiuyuan Ou, Yan Liu, Xiaobo Lei, Pei Li, Dan Mi, Lili Ren, Li Guo, Ruixuan Guo, Ting Chen, Jiaxin Hu, Zichun Xiang, Zhixia Mu, Xing Chen, Jieyong Chen, Keping Hu, Qi Jin, Jianwei Wang, Zhaohui Qian

**Affiliations:** 1 NHC Key laboratory of Systems Biology of Pathogens, Institute of Pathogen Biology, Chinese Academy of Medical Sciences and Peking Union Medical College, 100176 Beijing, China; 2 Institute of Medicinal Plant Development (IMPLAD), Chinese Academy of Medical Sciences (CAMS) and Peking Union Medical Collage (PUMC), 151 Malianwa Road North, Haidian District, 100193 Beijing, China; 3 Hengshui Third People’s Hospital, Heibei, China

**Keywords:** Antibodies, SARS virus, Virus-host interactions, Viral infection

## Abstract

Since 2002, beta coronaviruses (CoV) have caused three zoonotic outbreaks, SARS-CoV in 2002–2003, MERS-CoV in 2012, and the newly emerged SARS-CoV-2 in late 2019. However, little is currently known about the biology of SARS-CoV-2. Here, using SARS-CoV-2 S protein pseudovirus system, we confirm that human angiotensin converting enzyme 2 (hACE2) is the receptor for SARS-CoV-2, find that SARS-CoV-2 enters 293/hACE2 cells mainly through endocytosis, that PIKfyve, TPC2, and cathepsin L are critical for entry, and that SARS-CoV-2 S protein is less stable than SARS-CoV S. Polyclonal anti-SARS S1 antibodies T62 inhibit entry of SARS-CoV S but not SARS-CoV-2 S pseudovirions. Further studies using recovered SARS and COVID-19 patients’ sera show limited cross-neutralization, suggesting that recovery from one infection might not protect against the other. Our results present potential targets for development of drugs and vaccines for SARS-CoV-2.

## Abstract (web summary)

SARS-CoV-2 has spread globally. Here, the authors characterize the entry pathway of SARS-CoV-2, show that the SARS-CoV-2 spike protein is less stable than that of SARS-CoV, and show limited cross-neutralization activities between SARS-CoV and SARS-CoV-2 sera.

## Introduction

Coronaviruses (CoVs) infect human and animals and cause varieties of diseases, including respiratory, enteric, renal, and neurological diseases^1^. They are classified into four genera, alpha-CoV, beta-CoV, gamma-CoV, and delta-CoV^2^. Since beginning of this century, there have already been three zoonotic outbreaks of beta-CoVs. In 2002–2003, severe acute respiratory syndrome coronavirus (SARS-CoV)^3,4^, a lineage B beta-CoV, emerged from bat and palm civet^5,6^, and infected over 8000 people and caused about 800 deaths^7^. In 2012, Middle East respiratory syndrome coronavirus (MERS-CoV), a lineage C beta-CoV, was discovered as the causative agent of a severe respiratory syndrome in Saudi Arabia^8^, currently with 2494 confirmed cases and 858 deaths^9^, it remains endemic in Middle East, and dromedary camel is considered as the zoonotic reservoir host of MERS-CoV. At the end of 2019, a novel coronavirus, named SARS-CoV-2, was found in patients with severe pneumonia in Wuhan, China^10–12^. Viruses were isolated from patients and sequenced. Phylogenetical analysis revealed that it is a lineage B beta-CoV and closely related to a SARS-like (SL) CoV, RaTG13, discovered in a cave of Yunnan, China, in 2013^13^. They share about 96% nucleotide sequence identities, suggesting that SARS-CoV-2 might have emerged from a Bat SL-CoV. However, the intermediate host or whether there is an intermediate host remains to be determined.

**Fig. 1:**
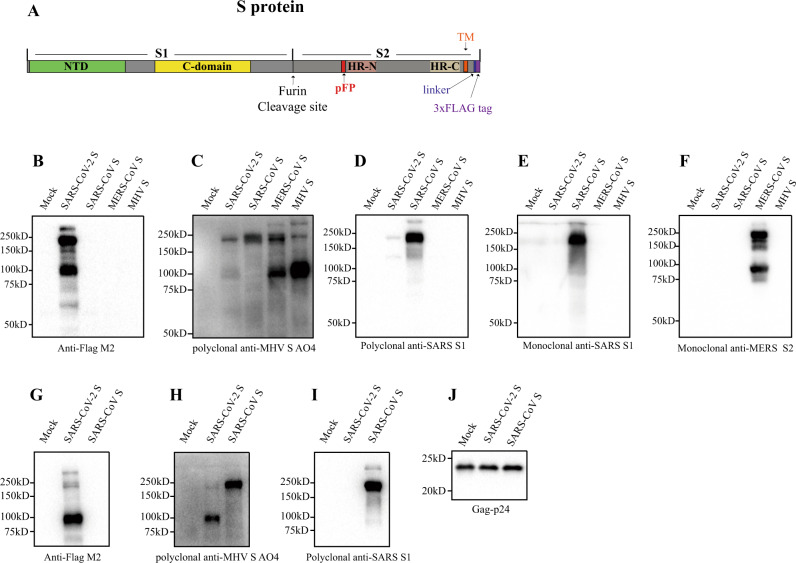
Incorporation of SARS-CoV-2 S protein into pseudovirions. **a** Diagram of full-length SARS-CoV-2 S protein with a 3xFLAG tag. S1, receptor-binding subunit; S2, membrane fusion subunit; TM, transmembrane domain; NTD, N-terminal domain; pFP, potential fusion peptide; HR-N, heptad repeat-N; HR-C, heptad repeat-C; **b**–**f** Detection of CoVs S protein in cells lysate by western blot. Mock, 293T cells transfected with empty vector. **b** Mouse monoclonal anti-FLAG M2 antibody; **c** Polyclonal goat anti-MHV-A59 S protein antibody AO4. **d** Polyclonal rabbit anti-SARS S1 antibodies T62. **e** Mouse monoclonal anti-SARS S1 antibody. **f** Mouse monoclonal anti-MERS-CoV S2 antibody. **g**–**j** Detection of CoVs S protein in pseudovirions by western blot.Gag-p24 served as a loading control. **g** Anti-FLAG M2. **h** Polyclonal goat anti-MHV-A59 S protein antibody AO4. **i** Polyclonal rabbit anti-SARS S1 antibodies T62. **j** Polyclonal anti-Gag-p24 antibodies. uncleaved S protein, about 180 kDa; cleaved S protein, about 90 kDa. Experiments were done twice and one is shown. Source data are provided as a Source Data file.

CoV uses its spike glycoprotein (S), a main target for neutralization antibody, to bind its receptor, and mediate membrane fusion and virus entry. Each monomer of trimeric S protein is about 180 kDa, and contains two subunits, S1 and S2, mediating attachment and membrane fusion, respectively. In the structure, N- and C- terminal portions of S1 fold as two independent domains, N-terminal domain (NTD) and C-terminal domain (C-domain) (Fig. 1a). Depending on the virus, either NTD or C-domain can serve as the receptor-binding domain (RBD). While RBD of mouse hepatitis virus (MHV) is located at the NTD^14^, most of other CoVs, including SARS-CoV and MERS-CoV use C-domain to bind their receptors^15–19^. MHV uses mouse carcinoembryonic antigen related cell adhesion molecule 1a (mCEACAM1a) as the receptor^20^, and the receptors for SARS-CoV and MERS-CoV are human angiotensin-converting enzyme 2 (hACE2)^21^ and human dipeptidyl peptidase 4 (hDPP4)^22^, respectively. While S proteins of SARS-CoV-2 share about 76% and 97% of amino acid identities with SARS-CoV and RaTG13, respectively, the amino acid sequence of potential RBD of SARS-CoV-2 is only about 74% and 90.1% homologous to that of SARS-CoV and RaTG13, respectively. Recently, Zhou et al.^13^ reported that SARS-CoV-2 uses hACE2 as the receptor.

CoV S proteins are typical class I viral fusion proteins, and protease cleavage is required for activation of the fusion potential of S protein^23^. A two-step sequential protease cleavage model has been proposed for activation of S proteins of SARS-CoV and MERS-CoV^24,25^, priming cleavage between S1 and S2 and activating cleavage on S2’ site. Depending on virus strains and cell types, CoV S proteins may be cleaved by one or several host proteases, including furin, trypsin, cathepsins, transmembrane protease serine protease-2 (TMPRSS-2), TMPRSS-4, or human airway trypsin-like protease (HAT)^25–32^. Availability of these proteases on target cells largely determines whether CoVs enter cells through plasma membrane or endocytosis. However, whether any of these proteases could promote virus entry of SARS-CoV-2 remains elusive.

In this study, using a lentiviral pseudotype system, we determine cell type susceptibility, virus receptor, entry pathway, and protease priming for SARS-CoV-2, and identify several potential drug targets for SARS-CoV-2. We demonstrate limited cross-neutralization between convalescent sera from SARS and COVID-19 patients.

## Results

### Expression of SARS-CoV-2 S protein

To enhance expression of the S protein of SARS-CoV-2 in mammalian cells, a codon-optimized cDNA encoding the S protein and 3xFLAG tag was synthesized, and to facilitate incorporation of S protein into lentiviral pseudovirons, the last 19 amino acids containing an endoplasmic reticulum (ER)-retention signal from the cytoplasmic tail of the S protein was removed (Fig. 1a). The construct was named SARS-CoV-2 S. HEK293T cells were transfected with SARS-CoV-2 S plasmid and expression of SARS-CoV-2 S protein was determined by western blot. There were two major bands, 180 kDa, and 90 kDa, detected by mouse anti-FLAG M2 antibody (Fig. 1b, lane 2), reflecting the full-length and cleaved S proteins, respectively. The band above 250 kDa likely results from dimeric or trimeric S proteins. Consistent with our previous report^29^, MERS-CoV S protein was detected by polyclonal goat anti-MHV S antibodies AO4 (Fig. 1c). AO4 also detected SARS-CoV-2 and SARS-CoV S proteins, suggesting the presence of a conserved immunogenic epitope among all four different CoVs. This presumably linear epitope is likely in S2^29^. S1 subunits of SARS-CoV-2 and SARS-CoV share almost 64% in amino acid identities. Nevertheless, SARS-CoV-2 S protein was barely detected by rabbit polyclonal anti-SARS S1 antibodies T62 (Fig. 1d), suggesting that the major epitope(s) for T62 antibodies include non-conserved regions of S1. The SARS-CoV-2 S protein was not detected by either a monoclonal anti-SARS S1 antibody (Fig. 1e) or anti-MERS S2 antibody (Fig. 1f).

### Pseudovirion incorporation of SARS-CoV-2 S protein

The efficiency of SARS-CoV-2 S protein incorporation into pseudovirions was evaluated using monoclonal mouse anti-FLAG M2 antibody and polyclonal goat anti-MHV S antibody AO4. While majority of SARS-CoV S proteins incorporated into pseudovirons were full-length, at 180 kDa (Fig. 1h, i), most of SARS-CoV-2 S proteins on lentiviral pseudovirions were cleaved, about 90 kDa (Fig. 1g, h), likely reflecting presence of extra furin site (R682-R683-A684-R685, Fig. 1a) between S1 and S2 in SARS-CoV-2 S protein. Consistent with the results in cell lysate (Fig. 1d), SARS-CoV S protein, but not SARS-CoV-2 S protein, in pseudovirions was readily detected by using polyclonal rabbit anti-SARS S1 antibodies T62 (Fig. 1i).

### Human ACE2 is a receptor for SARS-CoV-2

**Fig. 2:**
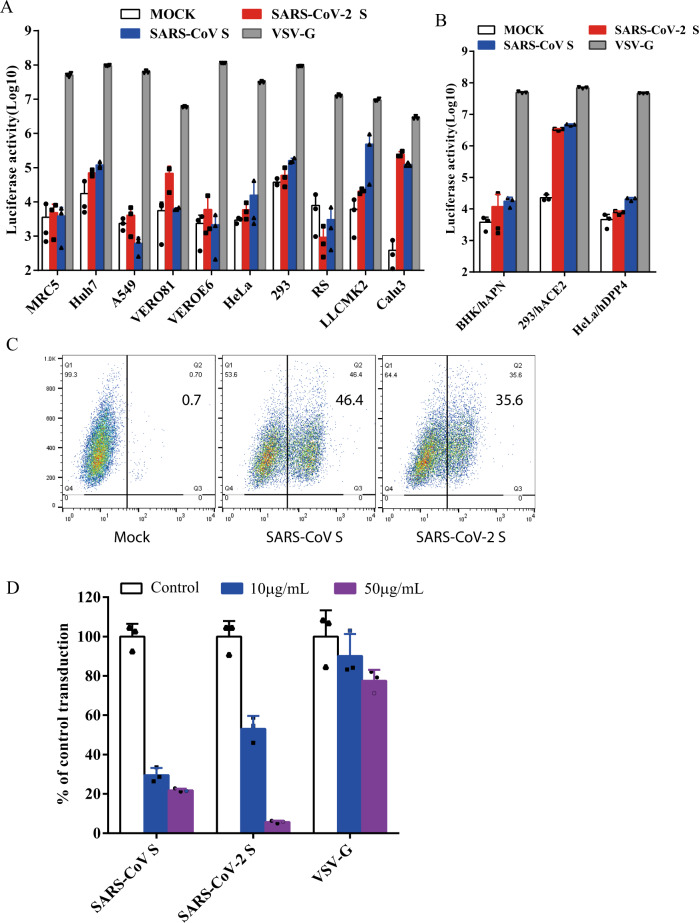
Entry and receptor of SARS-CoV-2 S pseudovirons. **a**, **b** Entry of SARS-CoV-2 S pseudovirions on indicated cell lines. Cells from human and animal origin were inoculated with SARS-CoV-2 S (red), SARS-CoV S (blue), or VSV-G (gray) pseudovirions. At 48 h post inoculation, transduction efficiency was measured according to luciferase activities. RS, *Rhinolophus sinicus* bat embryonic fibroblast; BHK/hAPN, BHK cells stably expressing hAPN, the hCoV-229E receptor; 293/hACE2, 293 cells stably expressing hACE2, the SARS-CoV receptor; HeLa/hDPP4, HeLa cells stably expressing hDPP4, the MERS-CoV receptor. Experiments were done in triplicates and repeated at least three times. One representative is shown with error bars indicating SEM. **c** Binding of SARS-CoV S and SARS-CoV-2 S proteins to soluble hACE2. HEK293T cells transiently expressing SARS-CoV and SARS-CoV-2 S proteins were incubated with the soluble hACE2 on ice, followed by polyclonal goat anti-hACE2 antibody. Cells were analyzed by flow cytometry. The experiments were repeated at least three times. **d** Inhibition of SARS-CoV-2 S pseudovirion entry by soluble hACE2. SARS-CoV S, SARS-CoV-2 S, or VSV-G pseudovirions were pre-incubated with soluble hACE2, then mixture were added to 293/hACE2 cells. Cells were lysed 40 h later and pseudoviral transduction was measured. Experiments were done twice and one representative is shown. Error bars indicate SEM of technical triplicates. Source data are provided as a Source Data file.

Next, we determined whether SARS-CoV-2 S pseudovirions were able to transduce human, monkey, and bat cells. VSV-G pseudovirons were used as a positive control, whereas bald particles with no spike proteins (mock) served as a negative control. As expected, all cell types were effectively transduced by VSV-G pseudovirons (Fig. 2a). Compared to mock control, SARS-CoV-2 S pseudovirions showed an over 500-fold increase in luciferase activities in Calu3 cells, at a level similar to SARS-CoV S pseudovirions (Fig. 2a). Huh7 and Vero 81 cells also gave about 10-fold increase in luciferase activities when transduced by SARS-CoV-2. Transduction of LLCMK2 cells was higher with SARS-CoV S pseudovirions than with SARS-CoV-2 S pseudovirions (Fig. 2a), suggesting that there might be some differences on virus entry on LLCMK2 cells mediated by S proteins between SARS-CoV-2 and SARS-CoV. We then investigated whether any known CoV receptors might be used by SARS-CoV-2 S protein as entry receptor. The SARS-CoV-2 S pseudovirons were used to transduce BHK cells stably expressing human aminopeptidase N (hAPN), the receptor for human CoV 229E, 293 cells stably expressing hACE2 (293/hACE2), the receptor for SARS-CoV, and HeLa cells stably expressing hDPP4 (HeLa/hDPP4), the receptor for MERS-CoV. While BHK/hAPN and HeLa/hDPP4 cells were not susceptible for the transduction of SARS-CoV-2 S pseudovirions, 293/hACE2 cells were highly transduced by SARS-CoV-2 S pseudovirions, consistent with hACE2 as the receptor for SARS-CoV-2^13^. We then determined whether SARS-CoV-2 S protein could directly bind to hACE2 protein. HEK 293T cells transiently expressing SARS-CoV-2 S protein were incubated with soluble hACE2 and analyzed by flow cytometry. As shown in Fig. 2c, SARS-CoV-2 S protein bound to soluble hACE2 at a level similar to SARS-CoV S protein, although the mean fluorescence intensity (MFI) for SARS-CoV-2 S protein was slightly lower than SARS-CoV S protein. To further investigate if hACE2 is the receptor for SARS-CoV-2, we performed inhibition experiments using soluble hACE2. Soluble hACE2 proteins were pre-incubated with SARS-CoV-2 S pseudovirons on ice for 1 h, then virus-protein mixture was added onto 293/hACE2 cells. Entry of SARS-CoV-2 S pseudovirions was significantly prevented by pre-incubation of soluble hACE2 at both 10 μg/ml and 50  μg/ml (Fig. 2d), further supporting the notion that hACE2 is the receptor and soluble hACE2 might be used as a therapeutic inhibitor against SARS-CoV-2 infection.

### The SARS-CoV-2 enters 293/hACE2 cells through endocytosis

**Fig. 3:**
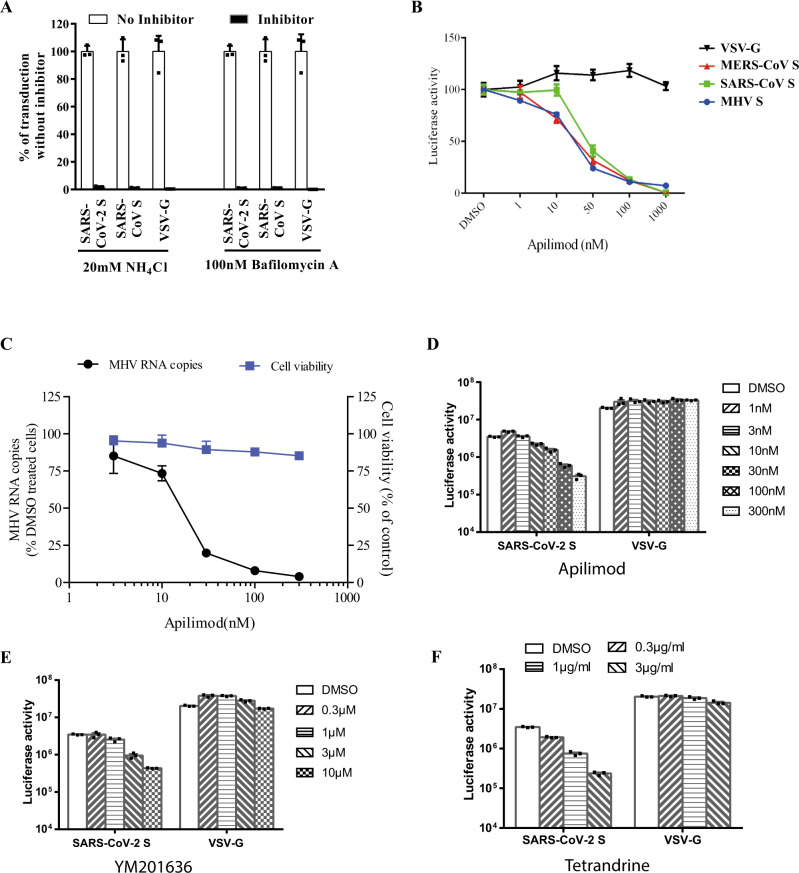
Endocytosis of SARS-CoV-2 S pseudovirions on 293/hACE2 cells. **a** Inhibition of entry of SARS-CoV-2 S pseudovirion on 293/hACE2 by lysosomotropic agents (20 mM NH_4_Cl and 100 nM bafilomycin A). **b** Inhibition of entry of SARS-CoV, MERS-CoV, and MHV S pseudovirions by a PIKfyve inhibitor apilimod. HeLa/mCEACAM, 293/hACE2, HeLa/hDPP4 cells were pretreated with different concentrations of apilimod and transduced with MHV S, SARS-CoV S, MERS-CoV S pseudovirions, respectively. The luciferase activity was measured 40 h post transduction. VSV-G pseudovirions were used as a control. Experiments were done in triplicates and repeated at least three times. One representative is shown with error bars indicating SEM. **c** Inhibition of MHV A59 infection by apilimod. The 17Cl.1 cells were pretreated with 3, 10, 30, 100, 300 nM apilimod for 30 min and infected by MHV A59 at MOI = 0.01. Viral infection and cell viability were determined by using qPCR and MTT assay, respectively. Experiments were done in triplicates and repeated at least three times. One representative is shown with error bars indicating SEM. **d**, **e** Inhibition of entry of SARS-CoV-2 S protein pseudovirions by apilimod, YM201636, and tetrandrine. HEK 293/hACE2 cells were pretreated with either apilimod (**d**), YM201636 (**e**), or tetrandrine (**f**), then inoculated with SARS-CoV-2 S pseudovirons in the presence of drug. The luciferase activity were measured 40 h post transduction. YM201636, PIKfyve inhibitor; tetrandrine, TPC2 inhibitor. The experiments were done in triplicates and repeated at least three times. One representative is shown with error bars indicating SEM of technical triplicates. Source data are provided as a Source Data file.

The majority of S proteins on SARS-CoV-2 S pseudovirions are cleaved (Fig. 1g, h). We next determined whether SARS-CoV-2 S pseudovirons entered cells through endocytosis or cell surface. HEK 293/hACE2 cells were treated with lysosomotropic agents, ammonia chloride and bafilomycin A, and their effect on virus entry was evaluated. Consistent with previous reports, 20 mM NH_4_Cl and 100 nM bafilomycin A decreased entry of SARS-CoV S and VSV-G pseudovirions by over 99%, compared to no treatment control. More than 98% reduction in transduction on 293/hACE2 cells by SARS-CoV-2 S pseudovirions was also shown when the cells were incubated with either NH_4_Cl or bafilomycin A (Fig. 3a), indicating that SARS-CoV-2 S pseudovirions enter 293/hACE2 cells mainly through endocytosis, despite that its spike proteins were cleaved.

### PIKfyve and TPC2 critical for SARS-CoV-2 entry

Phosphoinositides play many essential roles in endocytosis. Among them, one is phosphatidylinositol-3,5-bisphosphate (PI(3,5)P_2_), which regulates early endosome to late endosome dynamics^33,34^. Phosphatidylinositol 3-phosphate 5-kinase (PIKfyve) is the main enzyme synthesizing PI(3,5)P_2_ in early endosome. HEK 293/hACE2 cells were treated with apilimod, a potent inhibitor for PIKfyve^35^. Inhibition of PIKfyve by apilimod significantly reduced entry of SARS-CoV S pseudovirions on 293/hACE2 cells in a dose dependent manner (Fig. 3b), whereas it had no effect on entry of VSV-G pseudovirions, which occurs in early endosomes. Similar inhibitory effects were observed when HeLa/hDPP4 cells and HeLa cells stably expressing mouse carcinoembryonic antigen related cell adhesion molecule 1a (mCEACAM1a) (HeLa/mCEACAM1a) were treated with apilimod and transduced with MERS-CoV and MHV S pseudovirions (Fig. 3b), respectively. Moreover, infection of live MHV on 17Cl.1 cells was also strongly inhibited by apilimod treatment (Fig. 3c). No significant cell toxicity was observed on apilimod at any concentration tested (Fig. 3c). We then determined whether apilimod could inhibit entry of SARS-CoV-2 S pseudovirions on 293/hACE2. As expected, apilimod treatment inhibited entry of SARS-CoV-2 S pseudovirions in a dose dependent manner (Fig. 3d). Similar effects were shown when 293/hACE2 cells were treated with YM201636, another PIKfyve inhibitor (Fig. 3e). These results suggested that PIKfyve might be a potential general drug target for viruses that enter cells through endocytosis. Two pore channel subtype 2 (TPC2) and TRPML1 in lysosome are two major downstream effectors of PI(3,5)P_2_^36^. While blocking TPC2 activity by tetrandrine, an inhibitor for TPC2^37^, decreased entry of SARS-CoV-2 S pseudovirions (Fig. 3f), treatment of cells with 130, a TRPML1 inhibitor, had no effect (Supplementary Fig. 1), indicating that TPC2, not TRPML1, is important for SARS-CoV-2 entry.

### Effect of cathepsin inhibitors on SARS-CoV-2 entry

**Fig. 4:**
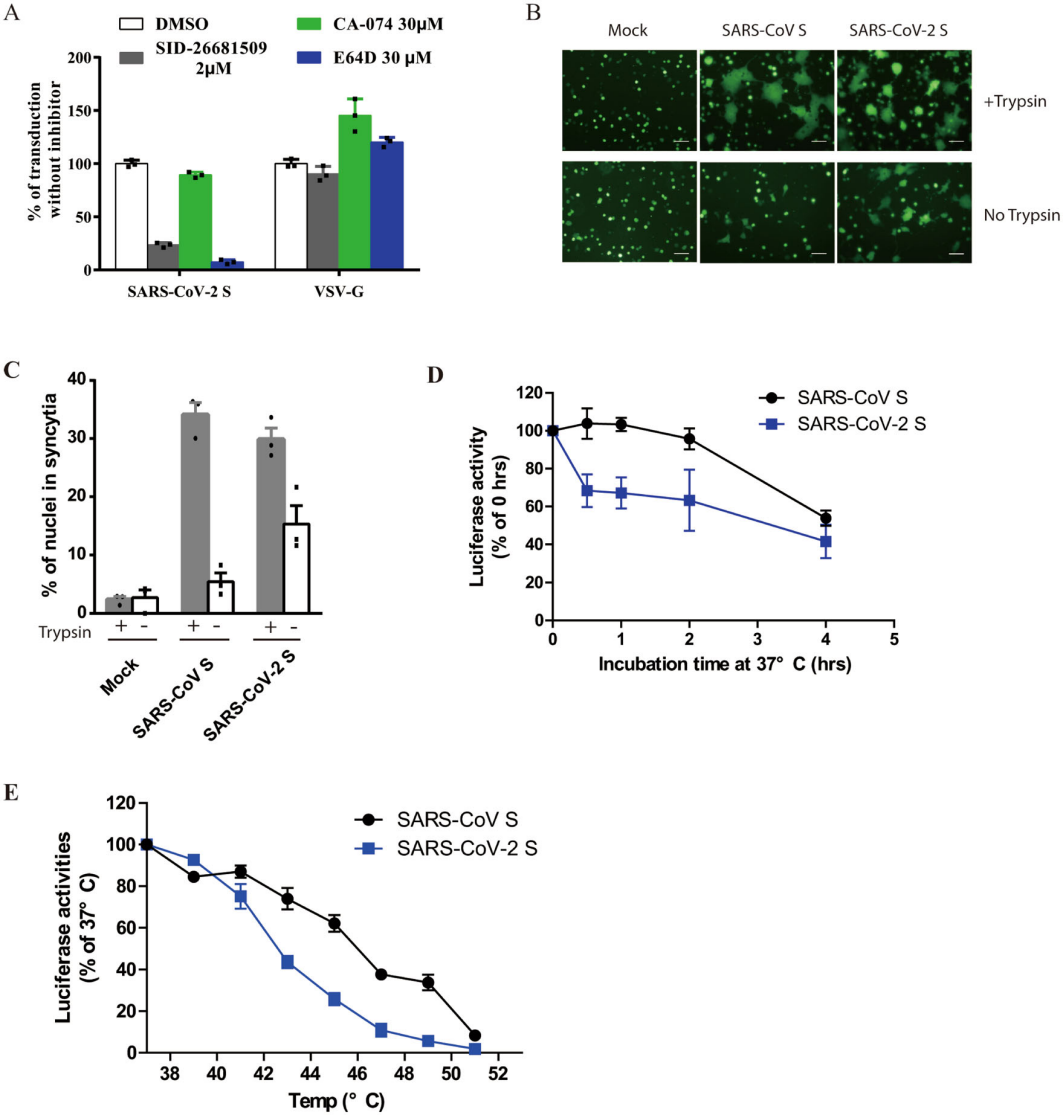
Activation of SARS-CoV-2 S protein by cathepsin and trypsin. **a** Effects of cathepsin inhibitors on entry of SARS-CoV-2 S pseudovirions on 293/hACE2 cells. HEK 293/hACE2 cells were pretreated with broad-spectrum cathepsin inhibitor E64D, cathepsin L-specific inhibitor (SID 26681509), or cathepsin B-specific inhibitor (CA-074) and then transduced with SARS-CoV-2 S and VSV-G pseudovirions. Pseudoviral transduction was measured at 40 h post inoculation. Experiments were done in triplicates and repeated at least three times. One representative is shown. Error bars indicate SEM of technical triplicates. **b** Cell–cell fusion mediated by SARS-CoV-2 S protein. HEK 293T cells were transiently expressing eGFP and either SARS-CoV-2 or SARS-CoV S protein were detached with either trypsin or EDTA, and co-cultured with 293/hACE2 or 293 cells for 4 h at 37 °C. The scale bar indicates 250 µm. **c** Quantitative analysis of syncytia in panel **b**. **d**, **e** Thermostability analysis of SARS-CoV-2 S protein. **d** SARS-CoV and SARS-CoV-2 S pseudovirons were incubated at 37 °C for the specified times (0 to 4 h) in the absence of serum, and then assayed on 293/hACE2 cells. The results from infection at 0 h were set as 100%, and the experiments were repeated four times, and means with standard deviations are shown. **e** SARS-CoV and SARS-CoV-2 S pseudovirions without serum were incubated at the indicated temperature (37 to 51 °C) for 2 h and then assayed on 293/hACE2 cells. The results are reported as the percentage of transduction at 37 °C. The experiments were repeated four times, and means with standard deviations are shown. Source data are provided as a Source Data file.

Protease activation on S protein is an important step for coronavirus entry, and cathepsins in lysosome have been shown to be critical for SARS-CoV and MERS-CoV entry through endocytosis. To investigate whether cathepsins are required for SARS-CoV-2 entry, HEK 293/hACE2 cells were treated with either a broad inhibitor for cathepsin B, H, L, and calpain (E64D), cathepsin L inhibitor (SID 26681509), or cathepsin B inhibitor (CA-074). VSV-G pseudovirions were used as a negative control, since virus entry mediated by VSV-G does not require protease activation. E64D treatment of 293/hACE2 cells reduced entry of SARS-CoV-2 S pseudovirions by 92.5%, indicating that at least one of cathepsins or calpain might be required for SARS-CoV-2 entry (Fig. 4a). While cathepsin B inhibitor treatment did not show any marked effect on virus entry, cathepsin L inhibition treatment decreased entry of SARS-CoV-2 S pseudovirions into 293/hACE2 by over 76% (Fig. 4a), suggesting that cathepsin L should be essential for priming of SARS-CoV-2 S protein in lysosome for entry into 293/hACE2 cells.

### Cell–cell fusion mediated by SARS-CoV-2 S protein

Type II membrane serine proteases (TMPRSS)-mediated cleavage can activate the fusion potential of SARS-CoV and MERS-CoV S protein and induce receptor-dependent syncytium formation^28,29,38,39^. To test whether SARS-CoV-2 S protein cleaved by these cellular proteases could trigger syncytium formation, we overlaid HEK 293T cells expressing SARS-CoV-2 S or control proteins on 293/hACE2 cells in presence or absence of proteases. Expression of TMPRSS 2, 4, 11 A, 11D, and 11E on 293/hACE2 cells enhanced SARS-CoV-2 S protein-mediated cell–cell fusion similarly to SASR-CoV S (Supplementary Fig. 2). Next we evaluated the role of trypsin. Consistent to our previous report^23^, addition of trypsin triggered syncytium formation on 293/hACE2 cells by SARS-CoV S protein after 4 h incubation (Fig. 4b, c). Large syncytia were formed at a level similar to SARS-CoV S proteins when SARS-CoV-2 S protein expressing 293T cells were added onto 293/hACE2 cells with trypsin. Of note, SARS-CoV-2, but not SARS-CoV, S proteins induced syncytia formation on 293/hACE2 cells even in the absence of trypsin (Fig. 4c), suggesting that SARS-CoV-2 S proteins could be triggered upon the receptor binding without exogenous protease priming or activation. During 2013–2016 Ebola virus outbreak, A82V and T544I mutations in glycoprotein (gp) of Ebola virus were linked to increase of virus transmissibility^40–42^. Further study showed that A82V and T544I mutation decreases the thermostability of gp while they increases virus infectivity in cell culture^42^. We then investigated the thermostability of SARS-CoV-2 S protein. Compared to SARS-CoV S protein, SARS-CoV-2 S protein was less stable, requiring significant shorter time and lower temperature to be inactivated (Fig. 4d, e). The native S protein is metastable, and there is an energy barrier that prevents it from undergoing conformational change before triggering, SARS-CoV-2 S protein might decrease its energy barrier by reducing its thermostability. This might contribute to high-transmission efficiency of SARS-CoV-2.

### Effect of anti-SARS S1 antibodies T62 on SARS-CoV-2 S protein

**Fig. 5:**
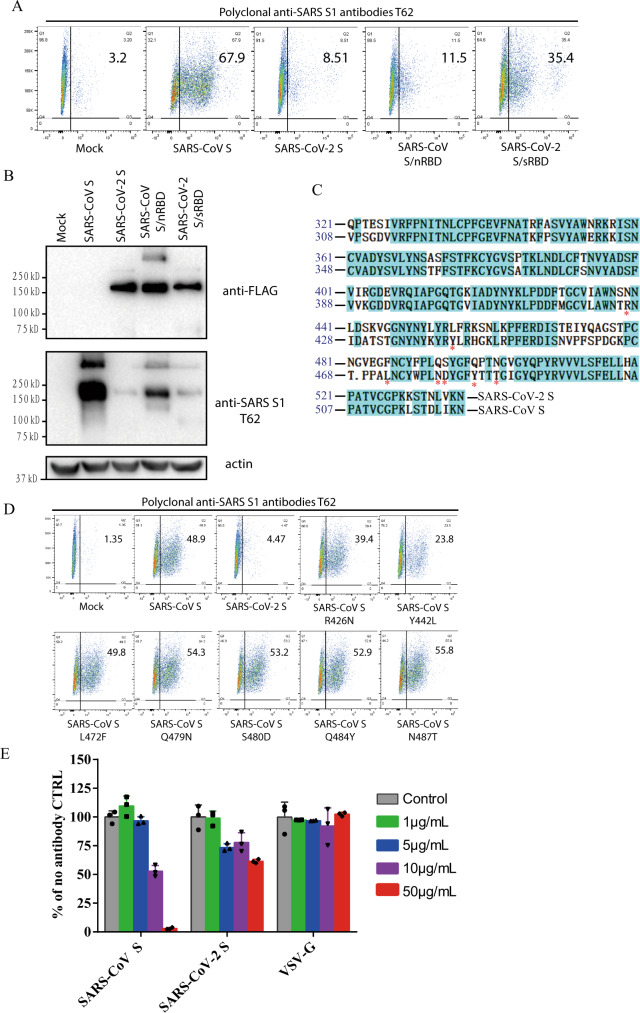
Characterization of polyclonal rabbit anti-SARS S1 antibodies T62. **a** Binding of polyclonal rabbit anti-SARS S1 antibodies T62 to SARS-CoV-2, SARS-CoV S, and chimeric S proteins. HEK293T cells transiently expressing either SARS-CoV-2 S, SARS-CoV S, SARS-CoV S/nRBD, or SARS-CoV-2 S/sRBD proteins were incubated with polyclonal rabbit anti-SARS-CoV S1 antibody T62 for 1 h on ice, followed by a FITC-conjugated secondary antibody, then cells were analyzed by flow cytometry. Experiments were done three times and one representative is shown. **b** Expression of SARS-CoV-2 S, SARS-CoV S, or chimeric S proteins on 293T cells. Cells from panel A were lyzed and blotted with anti-FLAG M2 antibody and polyclonal anti-SARS S1 antibody T62. **c** Amino acid sequence alignment of SARS-CoV and SARS-CoV-2 S RBDs. Stars (*) indicate the seven critical residues different between SARS-CoV-2 and SARS-CoV RBDs. **d** Binding of polyclonal rabbit anti-SARS S1 antibodies T62 to mutant SARS-CoV S proteins. **e** Neutralization of SARS-CoV-2 S and SARS-CoV S pseudovirions by polyclonal rabbit anti-SARS S1 antibody T62. Pseudovirons were pre-incubated with serially diluted polyclonal rabbit anti-SARS S1 antibodies T62 on ice, then virus-antibody mixture was added on 293/hACE2 cells. Pseudoviral transduction was measured 40 h later. Experiments were done in triplicates and repeated twice, and one representative is shown. Error bars indicate SEM of technical triplicates. Source data are provided as a Source Data file.

Since SARS-CoV-2 S reacted weakly with polyclonal rabbit anti-SARS S1 antibodies T62 in western blot (Fig. 1d), we investigated whether SARS-CoV-2 S protein in native conformation could be recognized by anti-SARS S1 antibodies T62. HEK293T cells transiently expressing SARS-CoV-2 S proteins were incubated with polyclonal anti-SARS S1 antibodies T62 and analyzed by flow cytometry. SARS-CoV S was used as a positive control. As expected, expression of SARS-CoV S proteins on 293T cell surface were readily detected by polyclonal anti-SARS S1 antibodies T62 (Fig. 5a). In contrast, only low level of binding of SARS-CoV-2 S protein to polyclonal rabbit anti-SARS S1 antibodies T62 was detected (Fig. 5a). To further delineate where the conformational epitopes for this polyclonal antibodies are located, two new constructs were generated, a SARS-CoV S protein backbone with RBD from SARS-CoV-2 (SARS-CoV S/nRBD) and a SARS-CoV-2 S protein backbone with RBD from SARS-CoV (SARS-CoV-2 S/sRBD). Replacement of SARS-CoV RBD with SARS-CoV-2 RBD (SARS-CoV S/nRBD) decreased binding of polyclonal antibodies T62 to S protein, whereas substitution of SARS-CoV-2 RBD with SARS-CoV RBD (SARS-CoV-2 S/sRBD) increased the affinity of S protein to polyclonal antibodies T62 (Fig. 5a). All chimera proteins were expressed at levels similar to SARS-CoV-2 S protein (Fig. 5b, top panel). These results suggest that at least one major conformational epitope for polyclonal antibodies T62 might be located in RBD where the sequences differ between SARS-CoV and SARS-CoV-2 (Fig. 5c). Of note, while SARS-CoV-2 S/sRBD bound to polyclonal antibodies T62 better than SARS-CoV S/nRBD in flow cytometry, the signal for SARS-CoV S/nRBD was stronger than SARS-CoV-2 S/sRBD in western blot by polyclonal antibodies T62, indicating that major linear epitopes of polyclonal antibodies T62 were located on SARS-CoV NTD. Among residues in SARS-CoV RBD critical for receptor binding and virus entry^43,44^, seven of them are different between SARS-CoV and SARS-CoV-2 RBDs (Fig. 5c). We then mutated each of them in SARS-CoV S protein to the corresponding residues in SARS-CoV-2 S. With exception of SARS-CoV mutant Y442L, which had reduced protein expression (Supplementary Fig. 3), binding of SARS-CoV S mutants to polyclonal rabbit anti-SARS S1 antibodies T62 was not affected (Fig. 5d). This suggests that these residues might not be direct binding sites for polyclonal antibodies T62. Next, we tested whether polyclonal rabbit anti-SARS S1 antibodies T62 could inhibit entry of SARS-CoV-2 S pseudovirions. CoV S protein pseudovirions were incubated with polyclonal antibodies T62 on ice for 1 h, and their transduction was measured according to luciferase activities. As expected, polyclonal antibodies T62 neutralized SARS-CoV S pseudovirion effectively in a dose dependent manner (Fig. 5e). In contrast, even at a concentration of 50 μg/ml, polyclonal antibodies T62 did not have marked effect on transduction by SARS-CoV-2 S proteins (Fig. 5e).

### Cross-neutralization of SARS and COVID-19 patient sera

**Fig. 6:**
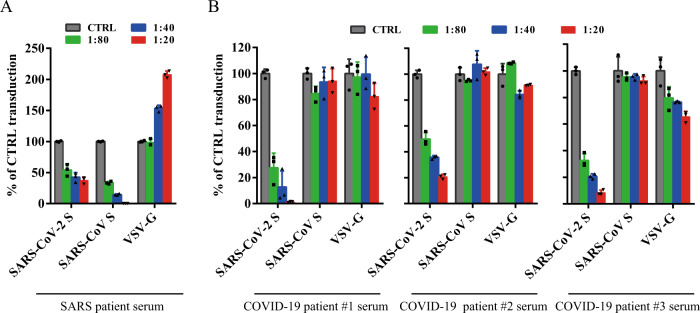
Limited cross-neutralization of SARS and COVID-19 sera. All sera were incubated on 56 °C for 30 min to eliminate complement. SARS-CoV S and SARS-CoV-2 S pseudovirons were pre-incubated with serially diluted SARS patient serum (**a**) or COVID-19 patient sera (**b**) for 1 h on ice and then added on 293/hACE2 cells. Pseudoviral transduction was measured 40 h later. Experiments were done in triplicates and repeated twice, and one representative is shown. Error bars indicate SEM of technical triplicates. Source data are provided as a Source Data file.

As rabbit polyclonal anti-SARS S1 antibodies did not show significant neutralization activity against SARS-CoV-2 S pseudovirions, we then asked whether sera from recovered SARS and COVID-19 patients could neutralize SARS-CoV S and SARS-CoV-2 S pseudovirions. We obtained sera from one recovered SARS patient and five recovered COVID-19 patients and tested their neutralization activities against transduction on 293/hACE2 cells by SARS-CoV and SARS-CoV-2 S pseudovirions. Serum from recovered SARS patient showed strong inhibition on transduction by SARS-CoV S pseudovirions, and it had modest neutralization activity against SARS-CoV-2 S pseudovirons (Fig. 6a and Table 1). In contrast, sera from all five COVID-19 patients neutralized SARS-CoV-2 S pseudovirions effectively, and none of them had an effect on transduction by SARS-CoV S pseudovirions (Fig. 6b and Table 1).

**Table 1:** Neutralization activities of antisera from SARS-CoV and COVID-19 patients.

Patient serum	SARS-CoV S pseudovirion	SARS-CoV-2 S pseudovirion
SARS-CoV patient	>80	40
COVID-19 patient #1	Not detected	>80
COVID-19 patient #2	Not detected	40
COVID-19 patient #3	Not detected	>80
COVID-19 patient #4	Not detected	>80
COVID-19 patient #5	Not detected	>80

SARS-CoV and SARS-CoV-2 S pseudovirions were pre-incubated with serially diluted patient sera for 1 h on ice, then virus-antibody mixture was added on 293/hACE2 cells. Cells were lysed 40 h later and pseudovirus transduction was measured. The highest dilution of the serum sample that decreased transduction by 50% or more was considered to be positive. Source data are provided as a Source Data file.

## Discussion

About 70% of the emerging pathogens infecting humans originate from animals, and CoVs are among the forefronts of these pathogens^45^. The newly emerged SARS-CoV-2 infects human and causes severe pneumonia, and as of 10 February, 2020, the current outbreak has spread to 25 countries with over 40,000 confirmed cases and 900 deaths^46^. However, little is known about its biology. Since the virus is categorized as a biosafety level 3 (BSL3) agent, according to WHO guideline, we developed a pseudotype system with S protein of SARS-CoV-2 to study virus entry in BSL2 settings. Understanding how SARS-CoV-2 enters cell will provide valuable information for virus pathogenesis, vaccine design, and drug target.

Utilizing this pseudotype system, we screened a panel of human and monkey cell lines for their susceptibility of SARS-CoV-2 S-mediated transduction. In line with SASR-CoV-2 causing respiratory infections in humans, we found that human lung cancer cell line Calu3 is highly susceptible to SARS-CoV-2 S-mediated entry. LLCMK2 cells, a rhesus monkey kidney epithelium cell line, exhibited different susceptibility to SARS-CoV S and SARS-CoV-2 S transduction, but the reasons for this are currently not known and require further investigation. Both S proteins use hACE2 as the receptor for binding and entry^13,21^, which we further confirmed by flow cytometry analysis and competitive inhibition experiment using soluble hACE2 in this study (Fig. 3b, c). While full-length S proteins of SARS-CoV-2 and SARS-CoV S share almost 76% identities in amino acid sequences, the NTDs show only 53.5% of homology. Of note, NTDs of different CoVs S proteins have been shown to bind different sugar. While NTD of MERS-CoV prefers α2,3-linked sialic acid over α2,6-linked sialic acid^47^, NTDs of human CoVs OC43 and HKU1 bind to 9-O-acetylated sialic acids^48,49^. No sugar binding has been reported for NTD of SARS-CoV. Whether or not NTD of SARS-CoV-2 binds to sugar and whether sugar binding of NTD affects virus entry remains to be determined.

Successful conformational changes of S proteins, leading to membrane fusion, not only require receptor binding, but also need appropriate protease activation. There is a furin site between S1 and S2 (amino acids 682-685, RRAR) subunits in SARS-CoV-2 S protein, similar to what happens in high-pathogenic influenza viruses. Whether the presence of this furin site has any effect on viral pathogenesis and virus spreading among humans remains to be determined. Although majority of SARS-CoV-2 S proteins in pseuodvirions were cleaved, endocytosis is the main entry pathway on 293/hACE2 cell. In our experiments, lysosomal cathepsin L, not B, appeared critical for SARS-CoV-2 S protein activation, similar to what was reported for SARS-CoV and MERS-CoV^28,29,50^. Trypsin could also activate SARS-CoV-2 S protein efficiently and induce large syncytium formation. To our surprise, we found that, even without trypsin, SARS-CoV-2 S protein could trigger syncytium formation on 293/hACE2 cells, a phenomena similar to what was observed in MHV S protein in certain aspect^51^. This led us to speculate that SARS-CoV-2 S protein is capable of triggering protease-independent and receptor-dependent syncytium formation. Such a mechanism might enhance virus spreading through cell–cell fusion and this might partially explain rapid progress of the diseases^10,11^. However, we have not excluded a potential role for other endogenous proteases in HEK293T cells for syncytium formation and we have also not evaluated entry into other cell types. This requires further research.

Recent studies showed that early to late endosome maturation is regulated by PI(3,5)P_2_, and inhibition of PIKfyve, the key enzyme synthesizing PI(3,5)P_2_, and TPC2, a downstream effector in lysosome, significantly reduced virus entry of MERS-CoV^52^. We confirmed this in this study, and further showed that blocking PIKfyve and TPC2 also strongly inhibited entry mediated by SARS-CoV-2 S protein, indicating that PI(3,5)P_2_ pathway might be considered as potential general drug target for CoV infection.

CoV S protein is one of the key components determining virus virulence, tissue tropism and host range, and it is also a main target for neutralizing antibodies and vaccine design. Although the S proteins of SARS-CoV-2 and SARS-CoV are highly homologous, polyclonal rabbit anti-SARS S1 antibodies T62 did not bind to SARS-CoV-2 S protein well, and poorly neutralized SARS-CoV-2 S protein-mediated virus entry. Further analysis reveals that major immune-epitopes for T62 antibodies likely lie in the region of RBD (Fig. 4a). We further evaluated cross-neutralization of SARS-CoV and SARS-CoV-2 using convalescent sera from SARS and COVID-19 patients. We only saw moderate cross-neutralization activities between SARS-CoV-2 and SARS-CoV convalescent sera, suggesting that those previously recovered from SARS-CoV infection may not be fully protected against SARS-CoV-2 infection, and vice versa.

While this manuscript was under review, Hoffmann et al.^53^ reported that human ACE2 is the entry receptor of SARS-CoV-2, and that serine protease TMPRSS2 is important for SARS-CoV-2 S activation, consistent with our findings. In agreement with our work, sera from convalescent SARS patients neutralized SARS-CoV-2 S pseudovirion entry with lower efficiency than SARS-CoV S pseudovirion entry, showing moderate cross-neutralization.

In conclusion, we demonstrated that SARS-CoV-2 S protein entry on 293/hACE2 cells is mainly mediated through endocytosis, and that PIKfyve, TPC2, and cathepsin L are critical for virus entry. We further found that SARS-CoV-2 S protein could trigger syncytia in 293/hACE2 cells independent of exogenous protease. Finally, there was limited cross-neutralization activity between convalescent sera from SARS and COVID-19 patients. Our findings provide potential targets for development of drugs and vaccines against this newly emerging lineage B beta-CoV.

## Methods

### Constructs and plasmids

Codon-optimized cDNA (sequence shown in Supplementary Table 2) encoding SARS-CoV-2 S glycoprotein (QHU36824.1) with C-terminal 19 amino acids deletion was synthesized and cloned into eukaryotic cell expression vector pCMV14-3×Flag between the *Hind* III and *BamH* I sites. Plasmids encoding SARS-CoV S glycoprotein, MERS-CoV S glycoprotein and MHV S glycoprotein (sequences shown in Supplementary Table 2) were constructed by inserting DNA fragment encoding codon-optimized SARS-CoV S protein (AAP13441.1) lacking the last 19 amino acids (aa), MERS-CoV S protein (AFS88936.1) lacking the last 16 aa but with a FLAG tag at the C terminus, or full-length MHV S protein (AAU06356.1) into pcDNA3.1between *BamH* I and *Not* I sites, respectively. The VSV-G encoding plasmid and lentiviral packaging plasmid psPAX2 were obtained from Addgene (Cambridge, MA). The pLenti-GFP lentiviral reporter plasmid that expresses GFP and luciferase was generously gifted by Fang Li, Duke University. All primers used in this study are listed in Supplementary Table 1.

### Cell lines

Human embryonic kidney cell line 293 (#CRL-1573) and 293T expressing the SV40 T-antigen (#CRL-3216), human airway epithelial cell line Calu3 (#HTB-55), human alveolar epithelial cell line A549 (#CCL-185), human fibroblasts derived from lung tissue MRC5 (#CCL-171), African green monkey kidney cell line Vero E6 (#CRL-1586) and Vero 81 (#CCL-81), and human cervical carcinoma cell line HeLa(#CCL-2) were obtained from ATCC (Manassas, VA, USA). Human carcinoma cell line derived from hepatocyte Huh7 was kindly provided by Dr. Wei Yang (Chinese Academy of Medical Sciences, Beijing, China). Bat embryo fibroblast cells RS were isolated mid-gestation fetuses from *Rhinolophus sinicus* bat. HEK 239 cells stably expressing recombinant human ACE2 (293/hACE2), baby hamster kidney fibroblasts stably expressing recombinant human APN (BHK/hAPN), HeLa cells stably expressing recombinant human DPP4 (HeLa/hDPP4) were established in our lab by overexpression of these receptors. All above cells were maintained in Dulbecco’s MEM containing 10% fetal bovine serum and 100 unit penicillin, 100 μg streptomycin, and 0.25 μg Fungizone (1% PSF, Gibco) per milliliter. Rhesus monkeys kidney cell line LLC-MK2 (#CCL-7) from ATCC was maintained in Opti-MEM containing 10% FBS and 1%PSF.

### Antibodies and inhibitors

Broad-spectrum cysteine protease inhibitor E64D (#HY-100229), Cathepsin L-specific inhibitor SID 26681509 (#HY-103353), Cathepsin B-specific inhibitor CA-074 (#HY-103350), PIKfyve inhibitors apilimod (#HY-14644) and YM201636 (#HY-13228), were purchased from Med Chem Express (MCE, New Jersey, USA). Calcium channel blocker tetrandrine (#S2403) was purchased from Selleck Chemicals (Texas, USA). Endosome acidification inhibitors NH_4_Cl (#A9434) and bafilomycin A (#19-148) were purchased from Sigma-Aldrich (St. Louis, MO, USA). Rabbit polyclonal against SARS S1 antibodies (#40150-T62), mouse monoclonal against MERS-CoV S2 antibody (#40070-MM11), mouse monoclonal against SARS S1 antibody (#40150-MM02), rabbit polyclonal against HIV-1 Gag-p24 antibody (11695-RP01) were purchased from Sino Biological Inc. (Beijing, China). Goat polyclonal against human ACE2 antibody (#AF933) was purchased from R&D Systems (Minnesota, USA). Mouse monoclonal anti-FLAG M2 antibody was purchased from Sigma-Aldrich. Donkey Anti-Rabbit IgG (#711-035-152), Goat Anti-Mouse IgG (#115-035-146), and Rabbit Anti-Goat IgG (#305-035-003) were purchased from Jackson ImmunoResearch (West Grove, PA, USA). Alexa Fluor 488-conjugated goat anti-rabbit IgG, Alexa Fluor 488-conjugated goat anti-mouse IgG were purchased from ZSGB-Bio LLC (Beijing, China).

### Production of SARS-CoV-2 S pseudovirions and virus entry

Pseudovirions were produced by co-transfection 293T cells with psPAX2, pLenti-GFP, and plasmids encoding either SARS-CoV-2 S, SARS-CoV S, VSV-G, or empty vector by using polyetherimide (PEI). The supernatants were harvested at 40, 64 h post transfection, passed through 0.45 μm filter, and centrifuged at 800 × *g* for 5 min to remove cell debris. To transduce cells with pseudovirions, cells were seeded into 24-well plates and inoculated with 500 μl media containing pseudovirions. After overnight incubation, cells were fed with fresh media. About 40 h post inoculation, cells were lysed with 120 μl medium containing 50% Steady-glo (promega) at room temperature for 5 min. The transduction efficiency was measured by quantification of the luciferase activity using a Modulus II microplate reader (Turner Biosystems, Sunnyvale, CA, USA). All experiments were done in triplicates, and repeated at least twice or more.

### Detection of S protein of SARS-CoV-2 by western blot

The spike glycoprotein of SARS-CoV-2 in cells and on pseudovirions were detected by using western blot. Briefly, cells transfected with plasmids encoding spike glycoprotein of SARS-CoV-2, SARS-CoV, MERS-CoV, and MHV were lysed at 40 h post transfection by RIPA buffer (20 mM Tris-HCl pH 7.5, 150 mM NaCl, 1 mM EDTA, 0.1% SDS, 1% NP40, 1×protease inhibitor cocktail). To pellet down pseudovirions, the viral supernatants were centrifuged at 25,000 rpm for 2 h in a Beckman SW41 rotor at 4 °C through a 20% sucrose cushion, and virus pellets were resuspended into 30 μl RIPA buffer. The samples were boiled for 10 min and separated in a 10% SDS-PAGE gel and transferred to nitrocellulose filter membranes. After blocked by 5% milk, the membranes were blotted with primary antibodies, followed by incubated with horseradish peroxidase (HRP) conjugated secondary antibodies (1:5000) and visualized with Chemiluminescent Reagent (Bio-Rad). MHV S proteins were detected using polyclonal goat anti-MHV S antibody AO4 (1:2000); SARS-CoV S proteins were blotted with either polyclonal anti-SARS S1 antibodies T62 (1:2000) (Sinobiological Inc, Beijing, China) or mouse monoclonal against SARS S1 antibody MM02 (1:1000) (Sinobiological Inc, Beijing, China), MERS-CoV and SARS-CoV-2 S proteins were detected using mouse monoclonal anti-MERS S (1:1000) (Sinobiological Inc, Beijing, China) and anti-FLAG M2 antibody (1:1000) (Sigma, St. Louis, MO, USA), respectively.

### Effects of agents and inhibitors on pseudovirion entry

HEK 293/hACE2 cells were pretreated with either lysosomotropic agents (endosome acidification inhibitor NH_4_Cl and bafilomycin A; PIKfyve inhibitor apilimod and YM201636; calcium channel blocker tetrandrine) or cathepsin inhibitors (cathepsin L inhibitor SID26881509, cathepsin B inhibitor CA-074, and cathepsin inhibitor E64D) for 1 h at 37 °C, then spin-inoculated with pseudovirions in the presence of inhibitor at 1200 × *g* for 30 min. After overnight incubation, cells were fed with fresh medium without inhibitor. Cells were lysed at 48 h post inoculation and their luciferase activity was measured.

### Flow cytometric analysis of S protein expression

Briefly, 293T cells were transfected with 2 μg of plasmids encoding either SARS-CoV-2 S, SARS-CoV S or MERS-CoV S protein using PEI. Forty hours later, cells were detached by using PBS with 1 mM EDTA. After washing, cells were incubated with polyclonal rabbit anti-SARS S1 antibodies T62 (1:200 dilution) (Sinobiological Inc., Beijing, China) for 1 h on ice, followed by Alexa Fluor 488-conjugated goat anti-rabbit IgG (1:200) (ZSGB-Bio LLC, Beijing, China) for 1 h. Cells were then analyzed by flow cytometry.

### MTT assay

MTT assay was used to assess cell viability. Briefly, cells were seeded into a 96-well plate at a cell density of 3000 per well and allowed to adhere for 24 h, followed by treatment with serially diluted inhibitors for 12 h. Two microliters of 5 mg/ml MTT was added to the medium 24 h later and incubated for 4 h at 37 °C. After removing the culture medium, 100 µl of DMSO was added. The absorbance was measured at 570 nm using a microplate spectrophotometer (Multiskan FC, Thermo Scientific). All experiments were performed in triplicate, and the cell viability of cells was calculated as the ratio of each experimental condition to the control.

### Cell–cell fusion assay

HEK-293T cells were co-transfected with plasmids encoding CoV S glycoprotein and eGFP. For trypsin-dependent cell–cell fusion, cells were detached with trypsin (0.25%) at 40 h post transfection and overlaid on an 80% confluent monolayer of 293/hACE2 cells at a ratio of approximately one S-expressing cell to three receptor-expressing cells. For trypsin-independent cell–cell fusion, cells were detached with 1 mM EDTA and overlaid on 293/hACE2 cells. After 4 h incubation, images of syncytia were captured with a Nikon TE2000 epifluorescence microscope running MetaMorph software (Molecular Devices). All experiments were done in triplicate and repeated at least three times.

### Expression and purification of soluble human ACE2

DNA fragments encoding residues 19-615 of human hACE2 with N-terminal FLAG and 6×his tags were cloned between *Sal* I and *Hind* III of modified pFASTBac1 vector with gp67 signal peptide. The soluble receptors were expressed in High Five insect cells using the bac-to-bac system (Invitrogen) and purified using nickel affinity and ion-exchange chromatography.

### Soluble hACE2-binding assay

HEK-293T cells were transfected with plasmids encoding SARS-CoV-2 S, SARS-CoV S, or MERS-CoV S protein. Cells were detached with 1 mM EDTA 40 h post transfection, washed twice with 3% FBS in 1×PBS, and incubated with 5 μg/ml soluble hACE2 for 1 h on ice. After washing three times with 3% FBS in 1×PBS, cells were incubated with polyclonal goat anti-human ACE2 antibody (1:200) (R&D Systems, MN, USA) for 1 h, followed by FITC-conjugated rabbit anti-goat secondary antibody (1:500) (Jackson ImmunoResearch, PA, USA). After washing, cells were then analyzed by flow cytometry.

### Soluble hACE2 inhibition assay

Briefly, lentiviruses pseudotyped with SARS-CoV-2 S, SARS-CoV S or VSV-G were pre-incubated with serially diluted soluble hACE2 for 1 h on ice. The mixture were then added on 293/hACE2 cells, followed by centrifugation inoculation for 1 h at room temperature. Cells were fed with fresh medium 6 h later and lysed at 48 h post inoculation. Pseudoviral transduction was measured according to luciferase activities.

### Pseudovirus neutralization assay

SARS-CoV S, SARS-CoV-2 S, and VSV-G pseudovirions were pre-incubated with serially diluted either polyclonal rabbit anti-SARS S1 antibodies T62 or patient sera for 1 h on ice, then virus-antibody mixture was added onto 293/hACE2 cells in a 96-well plate. After 6 h incubation, the inoculum was replaced with fresh medium. Cells were lysed 40 h later and pseudovirus transduction was measured as previously described. Prior to experiments, patient sera were incubated at 56 °C for 30 min to inactivate complement.

### Serum collection

Five patients were hospitalized with pneumonia in Hengshui Third People’s Hospital, Hengshui, Heibei province, China, and their respiratory specimens were collected for coronavirus detection and were positive for SARS-CoV-2. After all patients recovered, their sera were collected right before discharge with informed consent. Serum from a recovered SARS patient was also collected at two years after recovery with informed consent.

### Reporting summary

Further information on research design is available in the Nature Research Reporting Summary linked to this article.

## Supplementary information

Supplementary Information

Reporting Summary

## Data Availability

The data supporting the findings of this study are available from the authors upon request. The source data underlying Figs. 1b–j, 2a, b, d, 3a–f, 4a, c–e, 5b, e and 6a, b and Table 1 and Supplementary Figs. 1 and 3 are provided as a Source Data file.

## Source data

Source Data
