# Supplementary material for: Characterization of spike glycoprotein of SARS-CoV-2 on virus entry and its immune cross-reactivity with SARS-CoV
Source: Nat Commun. 2020 Mar 27;11:1620. doi: 10.1038/s41467-020-15562-9 (PMC7100515; doi:10.1038/s41467-020-15562-9)
Supplement: Supplementary file 1 — Supplementary Information [file 41467_2020_15562_MOESM1_ESM.pdf]

Supplementary information

Characterization of spike glycoprotein of SARS-CoV-2 on virus entry and its immune cross-reactivity with SARS-CoV

Ou *et al.*

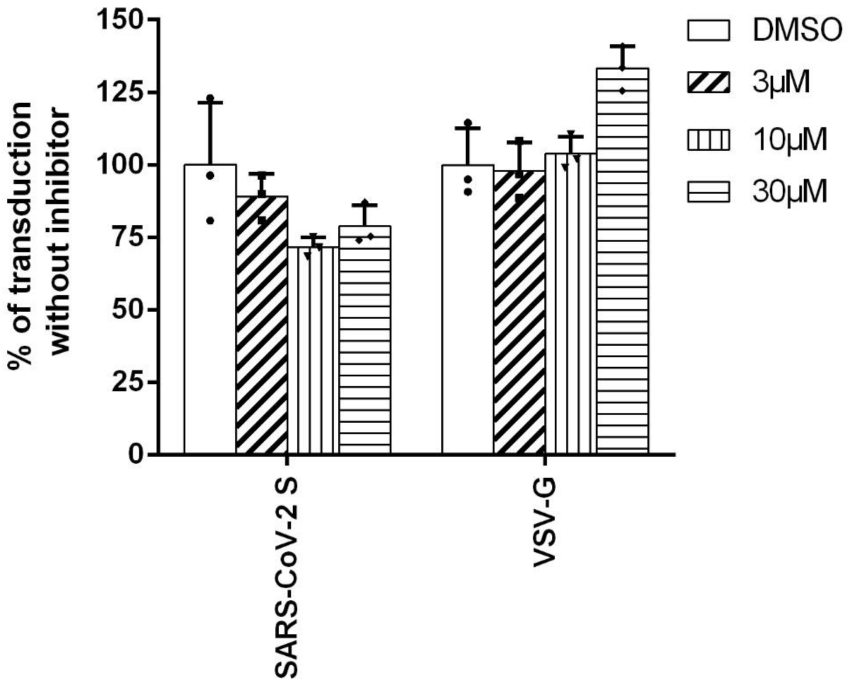

Supplementary Figure 1. Effect of entry of SARS-CoV-2 S pseudovirions on 293/hACE2 by TRPML1 inhibitor.

HEK 293/ACE2 cells were transduced with either SARS-CoV-2 S pseudovirions or VSV-G pseudovirions in the presence of 3, 10, 30μM 130. The luciferase activity were measured at 40 hrs post inoculation. The experiments were done triplicate and repeated three times.

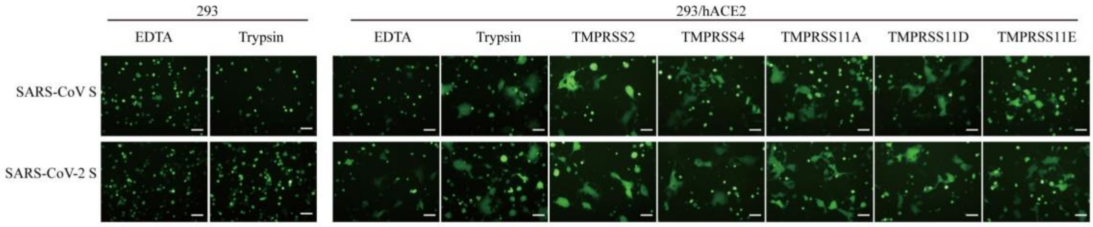

Supplementary Figure 2. Effect of type II membrane serine proteases (TMPRSS) on SARS-CoV-2 S protein mediated cell-cell fusion.

HEK-293T cells transiently expressing CoV S glycoprotein and eGFP were lifted with either trypsin (0.25%) or 1mM EDTA, then overlaid on 293/hACE2 cells transiently expressing type II membrane serine protease (TMPRSS) 2, 4, 11A, 11D, or 11E. After 4 hrs incubation, images of syncytia were captured.

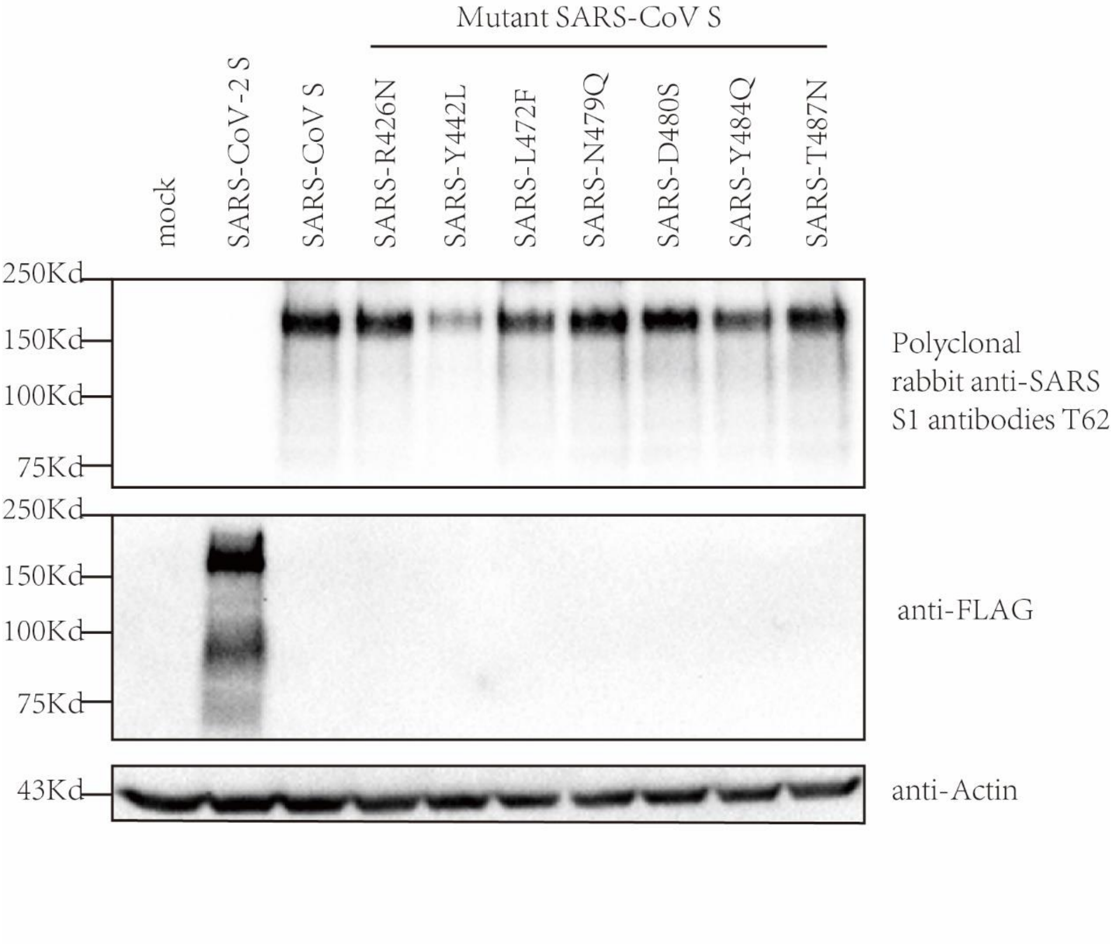

Supplementary Figure 3. Western blot analysis of expression of SARS-CoV mutant S proteins.

Top panel: polyclonal rabbit anti-SARS S1 antibodies T62; middle panel: anti-FLAG bottom panel: anti-actin.

Supplementary Table 1. Primers used in this study.

Constructs used in mutagenesis study	Forward primer sequence (5′-3′)	Reverse primer sequence (5′-3′)
SARS-CoV-2 S	CCGAAGCTTCACCATGTTCGTTTTCCTTGTTC	CCGGGATCCGCAACATGATCCGCAAGAGCAGC
SARS-CoV S-R426N	GTGCTGGCTTGGAACACTAACAACATTGATGCTACCTC	GAGGTAGCATCAATGTTGTTAGTGTTCCAAGCCAGCAC
SARS-CoV S-Y442L	CTACAACTACAAGTATCGTCTGCTGCGTCATGGTAAGCTG	CAGCTTACCATGACGCAGCAGACGATACTTGTAGTTGTAG
SARS-CoV S-L472F	CTTGCACTCCTCCAGCTTTCAACTGCTACTGGCCTC	GAGGCCAGTAGCAGTTGAAAGCTGGAGGAGTGCAAG
SARS-CoV S-N479Q	CTGCTACTGGCCTCTGCAGGATTACGGTTTCTACAC	GTGTAGAAACCGTAATCCTGCAGAGGCCAGTAGCAG
SARS-CoV S-D480S	CTACTGGCCTCTGAACTCTTACGGTTTCTACACC	GGTGTAGAAACCGTAAGAGTTCAGAGGCCAGTAG
SARS-CoV S-Y484Q	GAACGATTACGGTTTCCAGACCACTACCGGCATTGG	CCAATGCCGGTAGTGGTCTGGAAACCGTAATCGTTC
SARS-CoV S-T487N	GTTTCTACACCACTAACGGCATTGGCTACC	GGTAGCCAATGCCGTTAGTGGTGTAGAAAC

Supplementary Table 2. Codon-optimized DNA sequences of spike gene.

SARS-CoV-2 S	ATGTTCGTTTTCCTTGTTCTGTTGCCTCTCGTTAGTAGCCAATGCGTCAAC<br>CTTACTACTAGAACCCAGCTCCCTCCAGCATATACCAACTCTTTCACCAGG<br>GGCGTATATTACCCGGACAAAGTGTTCCGCTCAAGTGTGCTGCATTCTAC<br>GCAGGACCTTTTCTTGCCCTTTTTCAGTAATGTTACTTGGTTTCATGCTATC<br>CATGTGTCTGGAACTAACGGAACCAAGCGCTTTGACAACCCCGTCCTCC<br>CTTTCAACGATGGCGTGTACTTCGCTTCCACGGAAAAGTCAAACATAATT<br>CGCGGCTGGATCTTTGGTACAACACTCGACTCAAAGACGCAGAGCCTGC<br>TGATCGTTAATAACGCTACAAATGTTGTGATAAAGGTGTGTGAATTTCAGT<br>TCTGCAATGATCCCTTCCTGGGTGTGTACTACCATAAGAATAACAAGAGC<br>TGGATGGAATCCGAATTTAGGGTTTACAGTTCCGCTAACAACTGCACATT<br>CGAATACGTAAGCCAGCCATTTCTTATGGATCTTGAGGGCAAGCAAGGAA<br>ACTTCAAGAACTTGAGGGAGTTCGTGTTCAAAAATATCGACGGCTATTTT<br>AAGATATATAGCAAGCACACTCCAATAAACTTGGTGCGCGACCTGCCCCA<br>GGGATTCTCTGCTCTGGAGCCCCTGGTGGATCTGCCCATTGGAATAAACA<br>TAACTCGCTTTCAAACACTGCTCGCCCTGCATCGCAGTTACCTCACCCCT<br>GGTGATAGTAGTTCAGGATGGACAGCAGGAGCCGCCGCATACTACGTCG<br>GCTACCTGCAGCCTAGGACCTTCTTGCTGAAGTACAACGAGAACGGTAC<br>AATAACTGACGCTGTGGACTGCGCTCTGGACCCTCTGTCCGAGACGAAG<br>TGCACCCTGAAGAGCTTTACTGTTGAAAAAGGCATTTACCAAACCAGCA<br>ACTTCCGCGTCCAGCCAACCGAGAGCATCGTCAGATTTCCCAACATTACA<br>AATCTGTGTCCCTTCGGCGAGGTGTTCAACGCCACACGCTTCGCTTCAGT<br>GTACGCATGGAACCGCAAGCGCATATCTAACTGCGTCGCGGATTATTCTG<br>TCCTCTACAACTCCGCCTCTTTCTCCACCTTCAAGTGCTACGGAGTGTCA<br>CCGACTAAGCTGAACGATCTCTGCTTTACCAACGTCTACGCGGACTCCTT<br>CGTGATAAGAGGTGATGAAGTGAGACAAATAGCCCCAGGTCAGACTGGT<br>AAGATCGCAGATTACAACTACAAATTGCCTGATGATTTCACTGGTTGCGT<br>TATCGCGTGGAACTCTAATAACCTCGATTCTAAGGTCGGTGGTAACTACA<br>ATTACCTGTACCGCTTGTTTAGGAAGTCAAACCTGAAGCCTTTCGAGAGG<br>GATATTTCAACCGAAATCTATCAAGCGGGTTCAACACCGTGTAACGGTGT<br>GGAAGGATTTAACTGCTACTTCCCCCTGCAGTCTTACGGATTCCAGCCAA<br>CCAATGGCGTGGGTTACCAACCTTATCGCGTGGTGGTTCTGAGTTTCGAA<br>CTGTTGCACGCTCCCGCCACGGTATGCGGTCCCAAGAAGAGCACTAACT<br>TGGTGAAGAATAAGTGCGTGAATTTCAATTTCAATGGCCTCACTGGAACT<br>GGAGTGCTGACCGAATCCAATAAGAAGTTCTTGCCCTTCCAGCAGTTCGG<br>AAGAGACATTGCTGACACAACCGACGCGGTGCGCGATCCTCAGACTCTG<br>GAGATATTGGACATTACACCATGTTCTTTCGGCGGTGTGTCTGTCATTACT<br>CCGGGCACGAATACTAGCAACCAGGTAGCCGTGCTGTACCAAGACGTGA<br>ATTGCACAGAGGTTCCCGTCGCAATTCACGCTGACCAGCTGACCCCCAC<br>GTGGAGGGTTTACAGCACTGGTAGTAACGTCTTCCAGACGAGAGCCGGT<br>TGCTTGATCGGAGCGGAACATGTGAATAACTCCTACGAGTGCGACATCCC<br>CATCGGAGCCGGTATATGCGCCTCTTATCAGACACAAACTAACTCACCCA<br>GGAGAGCCCGCAGTGTGGCTTCTCAAAGCATTATAGCATACACTATGTCT<br>CTTGGTGCCGAAAATTCCGTGGCCTATTCTAACAATTCAATCGCCATCCCA

	ACCAACTTCACAATTAGCGTGACTACCGAAATACTGCCTGTGAGCATGAC<br>GAAAACCAGCGTAGACTGCACTATGTATATCTGTGGAGACTCCACTGAGT<br>GCTCCAACCTTCTCCTGCAGTACGGTAGCTTCTGTACCCAATTGAACCGC<br>GCCCTTACAGGCATCGCTGTTGAGCAAGATAAGAATACCCAGGAAGTTTT<br>TGCCCAGGTTAAGCAGATATACAAAACACCGCCCATTAAGGACTTCGGAG<br>GCTTCAACTTCTCTCAGATACTGCCTGACCCCTCCAAGCCATCAAAACGC<br>AGCTTCATTGAGGACCTCTTGTTCAACAAAGTGACTCTGGCTGATGCTGG<br>CTTCATTAAGCAGTACGGAGATTGCCTGGGAGATATTGCTGCCAGGGACC<br>TCATCTGCGCCCAGAAGTTTAATGGCCTGACAGTCTTGCCCCCACTTCTG<br>ACAGACGAGATGATTGCTCAGTACACATCTGCCCTCCTCGCTGGCACCAT<br>AACATCCGGATGGACATTTGGTGCTGGTGCTGCCCTCCAGATTCCCTTCG<br>CAATGCAGATGGCGTATCGCTTTAACGGCATCGGTGTCACACAAAACGTG<br>TTGTATGAGAACCAAAAGCTCATCGCTAACCAGTTTAATTCTGCTATTGGT<br>AAGATTCAGGACAGCCTGTCATCAACCGCGTCTGCCCTTGGTAAGTTGCA<br>GGACGTGGTGAACCAGAATGCTCAGGCTTTGAATACTCTGGTGAAGCAA<br>CTCTCTTCAAATTTCGGCGCTATCTCTTCTGTGTTGAACGACATCCTGAGT<br>CGCCTTGATAAGGTGGAAGCTGAAGTTCAAATTGATAGATTGATTACTGG<br>CAGGCTCCAGTCTTTGCAGACCTACGTTACACAGCAGCTGATTAGGGCGG<br>CTGAAATTAGAGCTTCCGCCAATCTGGCTGCAACCAAGATGTCCGAATGC<br>GTCCTGGGTCAGTCAAAGCGCGTTGACTTTTGTGGTAAAGGCTACCACCT<br>CATGTCATTTCCCCAGTCAGCACCTCACGGAGTAGTGTTCCTCCACGTCA<br>CCTACGTTCCAGCACAGGAAAAGAATTTTACCACTGCGCCGGCAATCTGT<br>CACGACGGTAAGGCACACTTCCCCCGCGAGGGCGTATTCGTGTCTAACG<br>GAACTCATTGGTTCGTCACACAGAGAAACTTCTATGAGCCTCAGATCATT<br>ACCACCGACAATACATTTGTGTCCGGTAACTGCGACGTTGTGATTGGAAT<br>CGTCAACAACACTGTGTACGATCCACTTCAGCCAGAACTGGATAGCTTCA<br>AGGAAGAATTGGACAAATATTTCAAAAATCACACTTCACCCGATGTGGAC<br>CTGGGTGACATTAGTGGTATCAATGCGTCCGTGGTCAATATTCAAAAAGA<br>GATTGACAGGCTCAACGAAGTGGCCAAGAACCTGAACGAAAGTCTTATC<br>GATCTGCAAGAATTGGGAAAGTATGAGCAGTACATCAAGTGGCCGTGGT<br>ACATTTGGTTGGGTTTTATCGCCGGTCTGATCGCCATCGTTATGGTTACCA<br>TTATGCTTTGCTGCATGACGAGCTGTTGCTCCTGTCTGAAGGGATGCTGC<br>TCTTGCGGATCATGTTGCAAGTTCGATGAAGACGATAGCGAACCAGTTCT<br>GAAGGGCGTCAAGCTGCATTACACA
SARS-CoV S	ATGTTCATCTTCCTGCTGTTCCTGACCCTGACCTCCGGCTCCGACCTGGA<br>CCGCTGCACCACCTTCGACGACGTGCAGGCTCCTAACTACACCCAGCAC<br>ACCTCCTCCATGCGCGGCGTGTACTACCCTGACGAGATCTTCCGCTCCGA<br>CACCCTGTACCTGACCCAGGACCTGTTCCTGCCCTTCTACTCCAACGTGA<br>CCGGCTTCCACACCATCAACCACACCTTCGGCAATCCCGTGATTCCCTTC<br>AAGGACGGCATCTACTTCGCCGCCACCGAGAAGTCCAACGTGGTGCGCG<br>GCTGGGTGTTCGGCTCCACCATGAACAACAAGTCCCAGTCCGTGATCATC<br>ATCAACAACTCCACCAACGTGGTGATCCGCGCCTGCAACTTCGAGCTGT<br>GCGACAATCCCTTCTTCGCCGTGTCCAAGCCCATGGGCACCCAGACCCA

	CACCATGATCTTCGACAACGCCTTCAACTGCACCTTCGAGTACATCTCCG<br>ACGCCTTCTCCCTGGACGTGTCCGAGAAGTCCGGCAACTTCAAGCACCT<br>GCGCGAGTTCGTGTTCAAGAACAAGGACGGCTTCCTGTACGTGTACAAG<br>GGCTACCAGCCCATCGACGTGGTGCGCGACCTGCCCTCCGGCTTCAACA<br>CCCTGAAGCCCATCTTCAAGCTGCCTCTGGGCATCAACATTACCAACTTC<br>CGCGCCATTCTGACCGCCTTCTCTCCCGCCCAGGACATCTGGGGCACCTC<br>CGCCGCCGCCTACTTCGTGGGCTACCTGAAGCCCACCACCTTCATGCTGA<br>AGTACGACGAGAACGGCACCATTACCGACGCCGTGGACTGCTCCCAGAA<br>CCCTCTGGCCGAGCTGAAGTGCTCCGTGAAGTCCTTCGAGATTGACAAG<br>GGCATTTACCAGACCTCCAACTTCCGCGTGGTGCCCTCCGGCGACGTGGT<br>GCGCTTTCCCAACATTACCAACCTGTGTCCCTTCGGCGAGGTGTTCAACG<br>CCACCAAGTTTCCCTCCGTGTACGCCTGGGAGCGCAAGAAGATTTCCAA<br>CTGCGTGGCCGACTACTCCGTGCTGTACAACTCCACCTTCTTCTCCACCT<br>TCAAGTGCTATGGCGTATCTGCCACCAAGCTGAACGATCTTTGCTTCTCC<br>AACGTGTACGCTGACTCCTTCGTGGTGAAAGGTGATGATGTTCGTCAGAT<br>TGCTCCTGGTCAGACTGGTGTGATTGCTGATTACAACTACAAGCTGCCTG<br>ATGATTTCATGGGTTGCGTGCTGGCTTGGAACACTCGTAACATTGATGCTA<br>CCTCCACTGGTAACTACAACTACAAGTATCGTTACCTGCGTCATGGTAAG<br>CTGCGTCCTTTCGAGCGTGATATTTCCAACGTGCCTTTCTCTCCTGATGGC<br>AAGCCTTGCACTCCTCCAGCTCTGAACTGCTACTGGCCTCTGAACGATTA<br>CGGTTTCTACACCACTACCGGCATTGGCTACCAGCCTTATCGTGTGGTTGT<br>GCTGTCTTTCGAGCTGCTGAACGCTCCTGCTACCGTGTGCGGTCCTAAGC<br>TGTCTACCGATCTGATTAAGAACCAGTGCGTGAACTTCAACTTCAACGGC<br>CTGACCGGCACCGGCGTGCTGACTCCCTCCTCCAAGCGCTTCCAGCCCTT<br>CCAGCAGTTCGGCCGCGATGTGTCCGATTTCACCGATTCCGTGCGCGATC<br>CCAAGACCTCCGAGATTCTGGATATTTCTCCCTGCGCCTTCGGCGGCGTG<br>TCCGTGATTACTCCCGGCACCAACGCCTCCTCCGAGGTGGCCGTGCTGTA<br>CCAGGATGTGAACTGCACCGATGTGTCCACCGCCATTCACGCCGATCAGC<br>TGACTCCCGCCTGGCGCATTTACTCCACCGGCAACAACGTGTTCCAGACC<br>CAGGCCGGCTGCCTGATTGGCGCCGAGCACGTGGATACCTCCTACGAGT<br>GCGATATTCCCATTGGCGCCGGCATTTGCGCCTCCTACCACACCGTGTCCC<br>TGCTGCGCTCCACCTCCCAGAAGTCCATTGTGGCCTACACCATGTCCCTG<br>GGCGCCGATTCCTCCATTGCCTACTCCAACAACACCATTGCCATTCCCAC<br>CAACTTCTCCATTTCCATTACCACCGAGGTGATGCCCGTGTCCATGGCCA<br>AGACCTCCGTGGATTGCAACATGTACATTTGCGGCGATTCCACCGAGTGC<br>GCCAACCTGCTGCTGCAGTACGGCTCCTTCTGCACCCAGCTGAACCGCG<br>CCCTGTCCGGCATTGCCGCCGAGCAGGATCGCAACACCCGCGAGGTGTT<br>CGCCCAGGTGAAGCAGATGTACAAGACTCCCACCCTGAAGTACTTCGGC<br>GGCTTCAACTTCTCCCAGATTCTGCCCGATCCTCTGAAGCCCACCAAGCG<br>CTCCTTCATTGAGGATCTGCTGTTCAACAAGGTGACCCTGGCCGATGCCG<br>GCTTCATGAAGCAGTACGGCGAGTGCCTGGGCGATATTAACGCCCGCGAT<br>CTGATTTGCGCGCAGAAGTTCAACGGCCTGACCGTGCTGCCTCCTCTGCT<br>GACCGATGATATGATTGCGGCGTACACCGCGGCGCTGGTGTCCGGCACCG<br>CCACCGCGGGCTGGACCTTCGGCGCGGGCGCGGCGCTGCAGATTCCCTT

	CGCGATGCAGATGGCGTACCGCTTCAACGGCATTGGCGTGACCCAGAAC<br>GTGCTGTACGAGAACCAGAAGCAGATTGCGAACCAGTTCAACAAGGCG<br>ATTTCCCAGATTCAGGAGTCCCTGACCACCACCTCCACCGCGCTGGGCA<br>AGCTGCAGGATGTGGTGAACCAGAACGCGCAGGCGCTGAACACCCTGG<br>TGAAGCAGCTGTCCTCCAACTTCGGCGCGATTTCCTCCGTGCTGAACGAT<br>ATTCTGTCCCGCCTGGATAAGGTGGAGGCGGAGGTGCAGATTGATCGCCT<br>GATTACCGGCCGCCTGCAGTCCCTGCAGACCTACGTGACCCAGCAGCTG<br>ATTCGCGCGGCGGAGATTCGCGCGTCCGCGAACCTGGCGGCGACCAAGA<br>TGTCCGAGTGCGTGCTGGGCCAGTCCAAGCGCGTGGATTTCTGCGGCAA<br>GGGCTACCACCTGATGTCCTTCCCTCAGGCGGCGCCTCATGGCGTGGTGT<br>TCCTGCACGTGACCTACGTGCCCTCCCAGGAGCGCAACTTCACCACCGC<br>GCCCGCGATTTGCCACGAGGGCAAGGCGTACTTCCCTCGCGAGGGCGTG<br>TTCGTGTTCAACGGCACCTCCTGGTTCATTACCCAGCGCAACTTCTTCTCT<br>CCTCAGATTATTACCACCGATAACACCTTCGTGTCCGGCAACTGCGATGT<br>GGTGATTGGCATTATTAACAACACCGTGTACGATCCTCTGCAGCCCGAGC<br>TGGATTCCTTCAAGGAGGAGCTGGATAAGTACTTCAAGAACCACACCTCT<br>CCCGATGTGGATCTGGGCGATATTTCCGGCATTAACGCCTCCGTGGTGAA<br>CATTCAGAAGGAGATTGATCGCCTGAACGAGGTGGCCAAGAACCTGAAC<br>GAGTCCCTGATTGATCTGCAGGAGCTGGGCAAGTACGAGCAGTACATTAA<br>GTGGCCTTGGTACGTGTGGCTGGGCTTCATTGCCGGCCTGATTGCCATTG<br>TGATGGTGACCATTCTGCTGTGCTGCATGACCAGTTGCTGCTCCTGCCTG<br>AAGGGCGCCTGCTCTTGCGGCTCTTGCTGCAAGTTCGACGAGGACGACT<br>CCGAGCCCGTGCTGAAGGGCGTGAAGCTGCACTACACC
SARS-<br>CoV-2<br>S/sRBD	ATGTTCGTGTTTCTGGTGCTGCTGCCCCTGGTGAGCTCCCAGTGCGTGAA<br>CCTGACCACAAGGACCCAGCTGCCACCTGCCTACACAAATAGCTTCACC<br>AGGGGCGTGTACTATCCTGACAAGGTGTTTCGCTCTAGCGTGCTGCACAG<br>CACACAGGATCTGTTTCTGCCATTCTTTTCCAACGTGACCTGGTTCCACG<br>CCATCCACGTGTCCGGCACAAATGGCACCAAGAGGTTCGACAATCCTGT<br>GCTGCCCTTCAACGATGGCGTGTACTTCGCCTCCACCGAGAAGTCTAACA<br>TCATCCGCGGCTGGATCTTTGGCACCACACTGGACAGCAAGACACAGTC<br>CCTGCTGATCGTGAACAATGCCACCAACGTGGTCATCAAGGTGTGCGAG<br>TTCCAGTTTTGTAATGATCCCTTCCTGGGCGTGTACTATCACAAGAACAAT<br>AAGTCTTGGATGGAGAGCGAGTTTAGAGTGTACTCCTCTGCCAACAATTG<br>CACATTTGAGTATGTGAGCCAGCCTTTCCTGATGGACCTGGAGGGCAAGC<br>AGGGCAATTTCAAGAACCTGCGGGAGTTCGTGTTTAAGAATATCGATGGC<br>TACTTTAAGATCTATAGCAAGCACACCCCCATCAACCTGGTGAGAGACCT<br>GCCTCAGGGCTTCTCCGCCCTGGAGCCACTGGTGGATCTGCCTATCGGCA<br>TCAACATCACAAGGTTTCAGACCCTGCTGGCCCTGCACCGCTCCTACCTG<br>ACACCAGGCGACAGCTCCTCTGGATGGACCGCAGGAGCAGCAGCCTACT<br>ATGTGGGCTACCTGCAGCCTAGGACCTTCCTGCTGAAGTATAACGAGAAT<br>GGCACAATCACCGACGCAGTGGATTGCGCCCTGGACCCACTGTCTGAGA<br>CAAAGTGTACCCTGAAGAGCTTTACAGTGGAGAAGGGCATCTACCAGAC<br>CTCTAATTTCCGGGTGGTGCCTAGCGGCGATGTGGTGAGATTTCCTAATAT<br>CACAAACCTGTGCCCATTTGGCGAGGTGTTCAACGCCACCAAGTTCCCA

	AGCGTGTACGCCTGGGAGCGCAAGAAGATCTCCAACTGCGTGGCCGACT<br>ACTCTGTGCTGTACAACTCCACATTCTTTTCTACCTTTAAGTGTTACGGCG<br>TGAGCGCCACCAAGCTGAATGACCTGTGCTTTAGCAACGTGTATGCCGAT<br>TCCTTCGTGGTGAAGGGCGACGATGTGAGGCAGATCGCACCAGGACAGA<br>CAGGCGTGATCGCAGACTACAATTATAAGCTGCCTGACGATTTCATGGGC<br>TGCGTGCTGGCCTGGAATACCCGCAACATCGATGCCACAAGCACCGGCA<br>ACTACAATTATAAGTACAGGTATCTGCGCCACGGCAAGCTGCGGCCTTTT<br>GAGAGAGACATCTCCAACGTGCCCTTCTCTCCTGATGGCAAGCCATGCAC<br>CCCACCCGCCCTGAATTGTTACTGGCCCCTGAACGACTACGGCTTCTATA<br>CCACAACCGGCATCGGCTACCAGCCATATAGAGTGGTGGTGCTGTCTTTT<br>GAGCTGCTGAATGCACCAGCAACAGTGTGCGGACCAAAGCTGAGCACC<br>GATCTGATCAAGAACCAGTGCGTGAACTTCAACTTCAACGGACTGACAG<br>GAACCGGCGTGCTGACCGAGAGCAACAAGAAGTTCCTGCCATTTCAGCA<br>GTTCGGCCGGGACATCGCCGATACAACCGACGCCGTGAGAGACCCACAG<br>ACACTGGAGATCCTGGATATCACCCCATGCAGCTTCGGCGGCGTGTCCGT<br>GATCACACCAGGAACAAATACCTCCAACCAGGTGGCCGTGCTGTACCAG<br>GACGTGAATTGTACCGAGGTGCCTGTGGCCATCCACGCCGATCAGCTGAC<br>ACCAACCTGGAGGGTGTATTCTACAGGAAGCAACGTGTTCCAGACCAGG<br>GCAGGATGCCTGATCGGAGCAGAGCACGTGAACAATTCTTACGAGTGCG<br>ACATCCCAATCGGCGCCGGCATCTGTGCCAGCTATCAGACACAGACCAAC<br>TCTCCAAGGAGAGCACGGAGCGTGGCAAGCCAGTCCATCATCGCCTACA<br>CCATGTCCCTGGGCGCCGAGAATTCTGTGGCCTATTCCAACAATTCTATCG<br>CCATCCCTACAAACTTCACCATCTCCGTGACAACCGAGATCCTGCCAGTG<br>TCTATGACAAAGACCAGCGTGGACTGCACAATGTACATCTGTGGCGATAG<br>CACCGAGTGCTCCAACCTGCTGCTGCAGTATGGCTCTTTTTGTACACAGC<br>TGAATAGAGCCCTGACCGGAATCGCAGTGGAGCAGGACAAGAACACAC<br>AGGAGGTGTTCGCCCAGGTGAAGCAGATCTACAAGACCCCTCCAATCAA<br>GGACTTTGGCGGCTTCAACTTCAGCCAGATCCTGCCAGATCCCAGCAAG<br>CCATCCAAGCGGTCTTTTATCGAGGACCTGCTGTTCAACAAGGTGACCCT<br>GGCCGATGCCGGCTTCATCAAGCAGTACGGCGATTGCCTGGGCGACATCG<br>CAGCAAGAGACCTGATCTGTGCCCAGAAGTTTAATGGCCTGACAGTGCT<br>GCCCCCTCTGCTGACCGATGAGATGATCGCCCAGTATACATCCGCCCTGC<br>TGGCCGGCACAATCACCTCTGGATGGACCTTCGGCGCAGGAGCCGCCCT<br>GCAGATCCCTTTTGCCATGCAGATGGCCTACAGGTTCAACGGCATCGGCG<br>TGACCCAGAATGTGCTGTATGAGAACCAGAAGCTGATCGCCAATCAGTTT<br>AACAGCGCCATCGGCAAGATCCAGGACAGCCTGAGCTCCACAGCATCCG<br>CCCTGGGCAAGCTGCAGGATGTGGTGAATCAGAACGCCCAGGCCCTGAA<br>TACCCTGGTGAAGCAGCTGTCTAGCAACTTCGGCGCCATCTCCTCTGTGC<br>TGAATGATATCCTGTCCAGGCTGGACAAGGTGGAGGCCGAGGTGCAGAT<br>CGACAGGCTGATCACAGGCCGCCTGCAGTCCCTGCAGACATACGTGACC<br>CAGCAGCTGATCAGGGCAGCAGAGATCAGAGCCTCTGCCAATCTGGCCG<br>CCACCAAGATGAGCGAGTGCGTGCTGGGACAGTCCAAGAGGGTGGACT<br>TTTGTGGCAAGGGCTACCACCTGATGTCTTTCCCTCAGAGCGCCCCACAC<br>GGCGTGGTGTTTCTGCACGTGACCTATGTGCCCGCCCAGGAGAAGAACT

	TCACAACCGCCCCTGCCATCTGCCACGATGGCAAGGCCCACTTTCCACG<br>GGAGGGCGTGTTCGTGAGCAACGGCACACACTGGTTTGTGACCCAGAG<br>AAATTTCTACGAGCCCCAGATCATCACAACCGACAATACATTCGTGTCCG<br>GCAACTGTGACGTGGTCATCGGCATCGTGAACAATACCGTGTACGATCCA<br>CTGCAGCCCGAGCTGGACTCCTTTAAGGAGGAGCTGGATAAGTATTTCAA<br>GAATCACACCTCTCCCGACGTGGATCTGGGCGACATCTCCGGCATCAATG<br>CCTCTGTGGTGAACATCCAGAAGGAGATCGACCGCCTGAACGAGGTGGC<br>CAAGAATCTGAACGAGAGCCTGATCGATCTGCAGGAGCTGGGCAAGTAC<br>GAGCAGTATATCAAGTGGCCTTGGTACATCTGGCTGGGCTTCATCGCCGG<br>CCTGATCGCCATCGTGATGGTGACAATCATGCTGTGCTGTATGACCAGCT<br>GCTGTTCCTGCCTGAAGGGCTGCTGTTCTTGTGGAAGCTGCTGTAAGTTC<br>GATGAAGACGATAGCGAACCAGTTCTGAAGGGCGTCAAGCTGCATTACA<br>CA
SARS-<br>CoV<br>S/nRBD	ATGTTCATCTTTCTGCTGTTCCTGACACTGACCAGCGGCTCCGACCTGGA<br>TAGGTGCACCACATTTGACGATGTGCAGGCCCCCAACTATACCCAGCACA<br>CAAGCTCCATGCGGGGCGTGTACTATCCTGATGAGATCTTCAGATCCGAC<br>ACCCTGTATCTGACACAGGACCTGTTCCTGCCCTTTTACTCTAATGTGACC<br>GGCTTCCACACAATCAATCACACCTTTGGCAACCCTGTGATCCCATTCAA<br>GGATGGCATCTACTTTGCCGCCACAGAGAAGAGCAACGTGGTGAGGGGC<br>TGGGTGTTCGGCTCCACCATGAACAATAAGTCTCAGAGCGTGATCATCAT<br>CAACAATTCCACCAACGTGGTCATCCGCGCCTGCAATTTTGAGCTGTGCG<br>ACAACCCATTCTTTGCCGTGTCTAAGCCCATGGGCACACAGACCCACACA<br>ATGATCTTTGATAATGCCTTCAACTGTACCTTTGAGTATATCTCCGATGCCT<br>TTTCTCTGGACGTGTCTGAGAAGAGCGGCAACTTCAAGCACCTGCGGGA<br>GTTCGTGTTTAAGAATAAGGACGGCTTCCTGTACGTGTATAAGGGCTACC<br>AGCCAATCGATGTGGTGAGAGACCTGCCCAGCGGCTTCAACACCCTGAA<br>GCCTATCTTTAAGCTGCCACTGGGCATCAATATCACAAACTTCAGGGCCAT<br>CCTGACCGCCTTTTCTCCTGCACAGGACATCTGGGGCACCAGCGCCGCA<br>GCATATTTCGTGGGCTACCTGAAGCCAACCACCTTCATGCTGAAGTATGAT<br>GAGAACGGCACCATCACAGACGCCGTGGATTGCAGCCAGAATCCTCTGG<br>CCGAGCTGAAGTGTTCCGTGAAGTCTTTCGAGATCGACAAGGGCATCTA<br>CCAGACATCCAACTTTCGGGTGCAGCCAACCGAGTCTATCGTGAGATTCC<br>CTAATATCACAAACCTGTGCCCATTCGGCGAGGTGTTTAATGCCACCCGC<br>TTTGCCTCCGTGTACGCCTGGAATCGGAAGAGAATCAGCAACTGCGTGG<br>CCGACTATTCCGTGCTGTACAACTCCGCCTCTTTCAGCACCTTTAAGTGCT<br>ATGGCGTGAGCCCCACAAAGCTGAATGACCTGTGCTTCACCAACGTGTA<br>CGCCGATTCCTTTGTGATCAGGGGCGACGAGGTGCGCCAGATCGCACCT<br>GGACAGACAGGCAAGATCGCCGACTACAACTATAAGCTGCCAGACGATT<br>TCACCGGCTGCGTGATCGCCTGGAATAGCAACAATCTGGATTCCAAAGTG<br>GGCGGCAACTACAATTATCTGTACAGGCTGTTCCGCAAGAGCAACCTGA<br>AGCCATTTGAGCGGGACATCAGCACAGAGATCTACCAGGCAGGATCCAC<br>CCCATGCAATGGAGTGGAGGGCTTCAACTGTTATTTTCCTCTGCAGTCCT<br>ACGGCTTCCAGCCAACCAATGGCGTGGGCTATCAGCCCTACAGAGTGGT<br>GGTGCTGTCTTTTGAGCTGCTGCACGCACCAGCAACAGTGTGCGGACCT

	AAGAAGAGCACCAATCTGGTGAAGAACAAGTGCGTGAACTTCAACTTCA<br>ACGGACTGACCGGAACAGGCGTGCTGACCCCCTCTAGCAAGAGGTTCCA<br>GCCTTTTCAGCAGTTCGGCCGCGACGTGTCTGATTTCACAGACAGCGTGC<br>GGGACCCTAAGACCTCCGAGATCCTGGATATCTCTCCATGCGCCTTTGGC<br>GGCGTGAGCGTGATCACCCCAGGAACAAACGCCTCCTCTGAGGTGGCCG<br>TGCTGTATCAGGATGTGAATTGTACAGACGTGTCCACCGCCATCCACGCC<br>GACCAGCTGACACCTGCCTGGAGGATCTACTCTACAGGCAACAACGTGT<br>TCCAGACCCAGGCAGGATGCCTGATCGGAGCAGAGCACGTGGATACCTC<br>CTATGAGTGCGACATCCCTATCGGCGCCGGCATCTGTGCCTCCTACCACA<br>CAGTGTCTCTGCTGCGCTCCACCTCTCAGAAGTCTATCGTGGCCTATACC<br>ATGAGCCTGGGCGCCGATAGCTCCATCGCCTACAGCAACAATACAATCGC<br>CATCCCAACCAACTTCAGCATCTCCATCACCACAGAAGTGATGCCCGTGA<br>GCATGGCCAAGACATCCGTGGACTGCAACATGTATATCTGTGGCGATTCC<br>ACCGAGTGCGCCAATCTGCTGCTGCAGTACGGCTCTTTTTGTACACAGCT<br>GAACAGAGCCCTGAGCGGAATCGCAGCAGAGCAGGACCGGAATACCAG<br>AGAGGTGTTCGCCCAGGTGAAGCAGATGTATAAGACCCCCACACTGAAG<br>TACTTTGGCGGCTTCAACTTTAGCCAGATCCTGCCAGATCCCCTGAAGCC<br>TACAAAGCGGTCCTTCATCGAGGACCTGCTGTTTAATAAGGTGACCCTGG<br>CCGATGCCGGCTTCATGAAGCAGTATGGCGAGTGCCTGGGCGATATCAAC<br>GCCAGAGACCTGATCTGTGCCCAGAAGTTTAATGGCCTGACAGTGCTGC<br>CCCCTCTGCTGACCGACGATATGATCGCCGCCTACACAGCCGCCCTGGTG<br>AGCGGCACCGCCACAGCAGGATGGACCTTCGGCGCAGGAGCCGCCCTG<br>CAGATCCCCTTCGCCATGCAGATGGCCTATCGGTTTAACGGCATCGGCGT<br>GACCCAGAATGTGCTGTACGAGAACCAGAAGCAGATCGCCAATCAGTTT<br>AACAAGGCCATCTCTCAGATCCAGGAGAGCCTGACCACAACCTCCACAG<br>CCCTGGGCAAGCTGCAGGACGTGGTGAATCAGAACGCCCAGGCCCTGA<br>ATACCCTGGTGAAGCAGCTGTCTAGCAACTTCGGCGCCATCTCCTCTGTG<br>CTGAATGATATCCTGTCCAGACTGGACAAGGTGGAGGCCGAGGTGCAGA<br>TCGATCGGCTGATCACAGGCAGACTGCAGAGCCTGCAGACCTACGTGAC<br>ACAGCAGCTGATCAGGGCAGCAGAGATCAGGGCATCCGCCAACCTGGCC<br>GCCACCAAGATGTCCGAGTGCGTGCTGGGCCAGTCTAAGAGGGTGGACT<br>TCTGTGGCAAGGGCTATCACCTGATGTCTTTTCCACAGGCAGCACCACAC<br>GGAGTGGTGTTCCTGCACGTGACCTACGTGCCCAGCCAGGAGCGCAACT<br>TTACAACCGCACCTGCAATCTGCCACGAGGGCAAGGCCTACTTCCCAAG<br>GGAGGGCGTGTTCGTGTTTAACGGCACATCCTGGTTTATCACCCAGCGCA<br>ATTTCTTTTCTCCCCAGATCATCACAACCGACAATACATTCGTGAGCGGCA<br>ACTGTGACGTGGTCATCGGCATCATCAACAATACCGTGTATGATCCCCTGC<br>AGCCTGAGCTGGACTCTTTCAAGGAGGAGCTGGATAAGTACTTTAAGAA<br>CCACACCAGCCCAGACGTGGATCTGGGCGACATCTCTGGCATCAATGCC<br>AGCGTGGTGAACATCCAGAAGGAGATCGACCGGCTGAATGAGGTGGCCA<br>AGAATCTGAACGAGAGCCTGATCGATCTGCAGGAGCTGGGCAAGTATGA<br>GCAGTACATCAAGTGGCCCTGGTACGTGTGGCTGGGCTTCATCGCCGGCC<br>TGATCGCCATCGTGATGGTGACAATCCTGCTGTGCTGTATGACCAGCTGC<br>TGTTCCTGCCTGAAGGGCGCCTGCTCTTGTGGAAGCTGCTGTAAGTTCGA

	CGAGGACGACTCCGAGCCCGTGCTGAAGGGCGTGAAGCTGCACTACACC
MERS-CoV S	ATGATTCACTCCGTGTTCCTCCTGATGTTCCTGCTCACTCCTACCGAAAGT<br>TATGTGGATGTGGGTCCCGATAGCGTCAAGTCCGCATGCATCGAGGTGGA<br>TATTCAGCAGACATTCTTTGACAAGACCTGGCCTCGGCCAATCGATGTCT<br>CCAAAGCCGACGGCATCATTTACCCTCAGGGACGCACCTATAGCAACATC<br>ACTATTACATACCAGGGCCTGTTCCCATATCAGGGCGACCACGGAGATATG<br>TACGTGTATTCTGCCGGACATGCTACTGGCACCACTCCTCAGAAACTGTT<br>CGTGGCAAATTACTCCCAGGACGTCAAGCAGTTCGCCAACGGTTTTGTG<br>GTCAGGATCGGCGCCGCTGCAAACTCTACCGGCACTGTGATCATTAGCCC<br>TTCTACTTCAGCCACAATCAGAAAAATTTACCCAGCTTTTATGCTGGGCTC<br>CAGCGTGGGAAACTTCTCCGATGGCAAGATGGGCCGTTTCTTTAATCACA<br>CTCTGGTCCTGCTCCCTGACGGATGCGGTACACTGCTCCGCGCTTTCTAC<br>TGTATCCTGGAGCCCAGGAGCGGAAATCACTGCCCTGCCGGTAACTCTTA<br>CACCTCATTCGCCACCTATCATACTCCAGCTACAGACTGTTCTGATGGCAA<br>TTATAACAGAAATGCCAGTCTGAACTCCTTCAAGGAATACTTTAATCTCCG<br>TAACTGCACATTCATGTACACCTATAATATCACTGAGGACGAAATTCTGGA<br>GTGGTTCGGTATCACACAGACCGCTCAGGGCGTGCACCTGTTTTCTTCAC<br>GCTACGTCGATCTCTATGGCGGAAACATGTTCCAGTTTGCCACTCTGCCC<br>GTGTACGACACAATCAAGTACTATTCCATCATTCCTCATAGCATCCGGTCT<br>ATTCAGTCAGACCGCAAGGCTTGGGCCGCTTTCTACGTGTATAAACTGCA<br>GCCTCTCACCTTCCTGCTCGATTTTTCCGTGGACGGATACATCCGCAGGG<br>CCATTGATTGCGGTTTCAACGACCTGAGCCAGCTCCACTGTAGTTATGAA<br>TCCTTCGATGTGGAGAGTGGTGTGTACTCCGTCAGTTCCTTTGAGGCTAA<br>GCCCAGTGGTTCCGTGGTCGAGCAGGCAGAAGGCGTGGAGTGCGACTTC<br>AGCCCTCTGCTCTCTGGCACACCCCCTCAGGTGTATAACTTCAAAAGGCT<br>GGTCTTTACCAACTGTAATTACAACCTGACTAAGCTGCTCAGCCTCTTCTC<br>TGTGAACGACTTTACCTGCTCACAGATCAGTCCCGCAGCCATTGCTTCAA<br>ATTGTTACAGCTCTCTGATCCTCGATTACTTCAGTTATCCTCTGTCCATGAA<br>AAGCGACCTCTCTGTGTCAAGTGCTGGCCCAATCTCCCAGTTTAATTACA<br>AGCAGAGCTTCTCTAACCCTACCTGCCTGATTCTCGCCACTGTGCCACAC<br>AACCTGACAACCATCACAAAGCCCCTCAAGTACAGCTACATCAACAAGT<br>GCTCAAGGCTGCTCAGTGACGATAGAACCGAAGTGCCACAGCTGGTCAA<br>TGCCAACCAGTACTCCCCATGTGTGAGCATCGTCCCCTCTACCGTGTGGG<br>AAGACGGAGATTACTATAGGAAGCAGCTGTCCCCCCTGGAGGGTGGAGG<br>ATGGCTGGTGGCATCAGGTAGTACAGTCGCCATGACCGAGCAGCTCCAG<br>ATGGGCTTCGGAATCACTGTGCAGTACGGCACTGATACAAATTCCGTCTG<br>CCCAAAGCTGGAATTTGCTAACGACACAAAAATCGCAAGCCAGCTGGGC<br>AATTGCGTGGAGTACTCCCTCTATGGAGTGAGCGGTCGTGGCGTCTTCCA<br>GAACTGTACTGCAGTGGGAGTCAGGCAGCAGAGGTTTGTGTACGATGCT<br>TATCAGAACCTGGTCGGCTACTATAGCGACGATGGAAATTACTATTGCCTC<br>CGCGCTTGTGTGTCAGTCCCCGTGAGTGTCATCTACGACAAGGAAACCA<br>AAACTCACGCAACCCTGTTCGGCTCTGTGGCCTGCGAGCATATTTCCAGC<br>ACTATGTCACAGTACTCCAGGAGCACAAGAAGTATGCTGAAGAGACGTG<br>ACTCCACATACGGACCCCTCCAGACCCCTGTGGGTTGCGTCCTGGGCCTC

	GTGAACTCTTCACTGTTCGTCGAAGATTGCAAGCTGCCTCTCGGACAGTC<br>CCTGTGTGCCCTCCCAGACACCCCCAGCACACTGACCCCACGTTCAGTG<br>CGGAGTGTCCCCGGCGAGATGCGTCTGGCATCTATCGCCTTCAATCACCC<br>AATTCAGGTGGATCAGCTGAACAGTTCCTACTTTAAGCTCTCTATCCCCAC<br>AAACTTCTCATTTGGCGTGACCCAGGAGTACATCCAGACTACAATTCAGA<br>AGGTGACCGTGGACTGCAAACAGTACGTGTGTAACGGATTCCAGAAATG<br>CGAACAGCTGCTCCGCGAGTACGGCCAGTTTTGTAGCAAGATCAATCAG<br>GCACTGCATGGCGCCAACCTCCGTCAGGACGATAGCGTGCGGAACCTGT<br>TCGCTTCTGTCAAGAGCTCTCAGTCAAGTCCTATCATTCCAGGTTTCGGT<br>GGCGACTTTAACCTGACCCTGCTCGAACCAGTGTCTATCTCAACTGGCAG<br>TCGCTCCGCTAGGAGCGCAATCGAGGATCTGCTCTTCGACAAGGTGACC<br>ATTGCCGACCCCGGATACATGCAGGGTTATGACGATTGCATGCAGCAGGG<br>ACCTGCCTCTGCTAGGGATCTGATCTGTGCTCAGTACGTGGCAGGTTATA<br>AGGTCCTGCCACCCCTCATGGACGTGAACATGGAAGCTGCATACACCTCC<br>AGCCTGCTCGGTTCCATCGCCGGAGTCGGTTGGACTGCTGGCCTGTCTTC<br>ATTCGCCGCTATCCCCTTTGCTCAGTCTATTTTCTACAGACTGAACGGCGT<br>GGGAATCACCCAGCAGGTCCTGTCAGAGAATCAGAAGCTCATTGCCAAT<br>AAATTCAACCAGGCCCTGGGAGCTATGCAGACTGGTTTTACCACTACAAA<br>CGAGGCCTTCCAGAAAGTGCAGGACGCAGTCAACAATAACGCACAGGC<br>CCTGAGCAAGCTCGCTTCCGAGCTGAGCAACACCTTCGGAGCAATCAGC<br>GCCTCTATTGGTGATATCATTCAGAGGCTGGACGTGCTCGAACAGGATGC<br>CCAGATCGACAGACTGATTAATGGCCGTCTGACCACTCTCAACGCTTTCG<br>TGGCACAGCAGCTGGTCCGGTCAGAAAGTGCAGCCCTGTCCGCCCAGCT<br>CGCTAAGGACAAAGTGAACGAGTGCGTCAAGGCTCAGTCTAAAAGATCA<br>GGATTTTGTGGTCAGGGCACCCACATCGTGAGCTTCGTGGTCAATGCCCC<br>AAACGGCCTGTACTTTATGCACGTCGGATACTATCCCTCAAACCATATCGA<br>GGTCGTCAGTGCTTACGGCCTGTGCGATGCTGCAAATCCTACAAACTGTA<br>TTGCCCCAGTGAACGGATACTTCATCAAGACCAACAACACTAGAATTGTG<br>GACGAATGGTCTTACACCGGCAGTTCCTTCTATGCACCCGAGCCTATCAC<br>TTCACTGAACACAAAGTACGTGGCCCCCCAGGTCACTTATCAGAATATCA<br>GTACAAACCTGCCTCCACCACTGCTCGGCAACTCCACCGGAATTGACTTC<br>CAGGATGAACTGGACGAGTTCTTTAAGAATGTGTCCACAAGCATCCCTAA<br>CTTTGGAAGCCTGACCCAGATTAACACAACCCTGCTCGATCTCACTTACG<br>AGATGCTGAGCCTCCAGCAGGTGGTCAAGGCTCTGAATGAATCTTACATC<br>GACCTGAAAGAGCTCGGTAATTATACATACTATAACAAGTGGCCCTGGTA<br>CATCTGGCTGGGTTTCATTGCAGGACTCGTGGCTCTGGCACTCTGCGTCT<br>TCTTTATCCTGTGCTGTACCGGATGCGGTACTAATTGTATGGGCAAGCTGA<br>AATGTAACCGCTGTTGCGACCGGTACGAGGAGTACGACCTGGAGCCCCA<br>CAAGGTGCACGTGCAC
MHV S	ATGCTGTTCGTGTTCATCCTGTTCCTGCCCAGCTGCCTGGGCTACATCGGC<br>GACTTCCGGTGCATCCAGCTGGTGAACAGCAACGGAGCAAACGTGAGC<br>GCACCAAGCATCAGCACCGAGACCGTGGAGGTGAGCCAGGGACTGGGC<br>ACCTACTACGTGCTGGACAGAGTGTACCTGAACGCCACCCTGCTGCTGA<br>CCGGCTACTACCCCGTGGACGGCAGCAAGTTCAGGAACCTGGCCCTGAC

	CGGCACCAACAGCGTGAGCCTGAGCTGGTTCCAGCCCCCTACCTGAGC<br>CAGTTCAACGACGGCATCTTCGCCAAGGTGCAGAACCTGAAGACCAGCA<br>CCCCCAGCGGCGCCACCGCCTACTTCCCCACCATCGTGATCGGCAGCCTG<br>TTCGGCTACACCAGCTACACCGTGGTCATCGAGCCCTACAACGGCGTGAT<br>CATGGCCAGCGTGTGCCAGTACACCATCTGCCAGCTGCCCTACACCGACT<br>GCAAGCCCAACACCAACGGCAACAAGCTGATCGGCTTCTGGCACACCGA<br>CGTGAAGCCCCCCATCTGCGTGCTGAAGCGCAACTTCACCCTGAACGTG<br>AACGCCGACGCCTTCTACTTCCACTTCTACCAGCACGGCGGCACCTTCTA<br>CGCCTACTACGCCGACAAGCCCAGCGCCACCACCTTCCTGTTCAGCGTGT<br>ACATCGGCGACATCCTGACCCAGTACTACGTGCTGCCCTTCATCTGCAAC<br>CCAACCGCAGGCAGCACCTTCGCACCAAGGTACTGGGTGACCCCACTGG<br>TGAAGAGACAGTACCTGTTCAACTTCAACCAGAAGGGCGTGATCACCAG<br>CGCCGTGGACTGCGCCAGCAGCTACACCAGCGAGATCAAGTGCAAGACC<br>CAGAGCATGCTGCCCAGCACCGGAGTGTACGAGCTGAGCGGATACACCG<br>TGCAGCCAGTGGGAGTGGTGTACAGGAGGGTGGCAAACCTGCCAGCATG<br>CAACATCGAGGAGTGGCTGACCGCACGGAGCGTGCCCAGCCCCCTGAAC<br>TGGGAGAGAAAGACCTTCCAGAACTGCAACTTCAACCTGAGCAGCCTGC<br>TGAGATACGTGCAGGCCGAGAGCCTGTTCTGCAACAACATCGACGCCAG<br>CAAGGTGTACGGCCGCTGCTTCGGCAGCATCAGCGTGGACAAGTTCGCC<br>GTGCCCCGGAGCAGACAGGTGGACCTGCAGCTGGGCAACAGCGGCTTC<br>CTGCAGACCGCCAACTACAAGATCGACACCGCCGCCACCAGCTGCCAGC<br>TGCACTACACCCTGCCCAAGAACAACGTGACCATCAACAATCACAACCC<br>CAGCAGCTGGAACAGGCGCTACGGCTTCAACGACGCCGGCGTGTTCGGC<br>AAGAACCAGCACGACGTGGTGTACGCCCAGCAGTGCTTCACCGTGCGGA<br>GCAGCTACTGCCCATGCGCACAGCCAGACATCGTGAGCCCATGCACCAC<br>CCAGACCAAGCCAAAGAGCGCCTTCGTGAACGTGGGCGACCACTGCGA<br>GGGACTGGGAGTGCTGGAGGACAACTGCGGAAACGCCGACCCCCACAA<br>GGGATGCATCTGCGCCAACAACAGCTTCATCGGCTGGAGCCACGACACC<br>TGCCTGGTGAACGACAGATGCCAAATCTTCGCCAACATCCTGCTGAACG<br>GCATCAACAGCGGCACCACCTGCAGCACCGACCTGCAGCTGCCCAACAC<br>CGAGGTGGTGACCGGCATCTGCGTGAAGTACGACCTGTACGGCATCACC<br>GGCCAGGGCGTGTTCAAGGAGGTGAAGGCCGACTACTACAACAGCTGG<br>CAGACCCTGCTGTACGACGTGAACGGCAACCTGAACGGCTTCAGGGACC<br>TGACCACCAACAAGACCTACACCATCCGCAGCTGCTACAGCGGCCGGGT<br>GAGCGCAGCATTCCACAAGGACGCACCAGAGCCAGCCCTGCTGTACAGA<br>AACATCAACTGCAGCTACGTGTTCAGCAACAACATCAGCAGGGAGGAGA<br>ACCCCCTGAACTACTTCGACAGCTACCTGGGATGCGTGGTGAACGCAGA<br>CAACAGGACCGACGAGGCCCTGCCAAACTGCGACCTGAGGATGGGAGC<br>AGGACTGTGCGTGGACTACAGCAAGAGCAGAAGGGCCCACCGCAGCGT<br>GAGCACCGGCTACAGACTGACCACCTTCGAGCCCTACACCCCCATGCTG<br>GTGAACGACAGCGTGCAGAGCGTGGACGGCCTGTACGAGATGCAGATCC<br>CCACCAACTTCACCATCGGCCACCACGAGGAGTTCATCCAGACCCGGAG<br>CCCCAAGGTGACCATCGACTGCGCCGCCTTCGTGTGCGGCGACAACACC<br>GCCTGCAGACAGCAGCTGGTGGAGTACGGCAGCTTCTGCGTGAACGTGA

	ACGCCATCCTGAACGAGGTGAACAACCTGCTGGACAACATGCAGCTGCA<br>GGTGGCCAGCGCACTGATGCAGGGAGTGACCATCAGCAGCAGGCTGCCA<br>GACGGAATCAGCGGCCCCATCGACGACATCAACTTCAGCCCCCTGCTGG<br>GATGCATCGGCAGCACCTGCGCAGAGGACGGAAACGGACCAAGCGCAA<br>TCCGCGGCCGGAGCGCCATCGAGGACCTGCTGTTCGACAAGGTGAAGCT<br>GAGCGACGTGGGCTTCGTGGAGGCCTACAACAACTGCACCGGCGGCCA<br>GGAGGTGAGAGACCTGCTGTGCGTGCAGAGCTTCAACGGCATCAAGGTG<br>CTGCCACCAGTGCTGAGCGAGAGCCAGATCAGCGGATACACCACCGGAG<br>CAACCGCTGCCGCAATGTTCCCACCCTGGAGCGCTGCCGCAGGCGTGCC<br>CTTCAGCCTGAGCGTGCAGTACAGGATCAACGGCCTGGGCGTGACCATG<br>AACGTGCTGAGCGAGAACCAGAAGATGATCGCCAGCGCCTTCAACAACG<br>CCCTGGGCGCCATCCAGGACGGCTTCGACGCCACCAACAGCGCCCTGGG<br>CAAGATCCAGAGCGTGGTGAACGCCAACGCCGAGGCCCTGAACAACCT<br>GCTGAACCAGCTGAGCAACAGGTTCGGAGCAATCAGCGCCAGCCTGCA<br>GGAGATCCTGACCCGGCTGGAGGCAGTGGAGGCAAAGGCACAGATCGA<br>CAGACTGATCAACGGCAGGCTGACCGCCCTGAACGCCTACATCAGCAAG<br>CAGCTGAGCGACAGCACCCTGATCAAGGTGAGCGCCGCCCAGGCCATCG<br>AGAAGGTGAACGAGTGCGTGAAGAGCCAGACCACCCGCATCAACTTCT<br>GCGGCAACGGCAACCACATCCTGAGCCTGGTGCAGAACGCCCCCTACGG<br>CCTGTACTTCATCCACTTCAGCTACGTGCCAATCAGCTTCACCACCGCAA<br>ACGTGAGCCCAGGACTGTGCATCAGCGGCGACAGGGGCCTGGCCCCAA<br>AGGCAGGCTACTTCGTGCAGGACGACGGCGAGTGGAAGTTCACCGGCA<br>GCAGCTACTACTACCCCGAGCCCATCACCGACAAGAACAGCGTGATCATG<br>AGCAGCTGCGCCGTGAACTACACCAAGGCCCCCGAGGTGTTCCTGAACA<br>CCAGCATCCCCAACCCTCCCGACTTCAAGGAGGAGCTGGACAAGTGGTT<br>CAAGAACCAGACCAGCATCGCCCCCGACCTGAGCCTGGACTTCGAGAAG<br>CTGAACGTGACCCTGCTGGACCTGACCTACGAGATGAACAGAATCCAGG<br>ACGCCATCAAGAAGCTGAACGAGAGCTACATCAACCTGAAGGAAGTGG<br>GCACCTACGAGATGTACGTGAAGTGGCCCTGGTACGTGTGGCTGCTGATC<br>GGCCTGGCCGGAGTGGCAGTGTGCGTGCTGCTGTTCTTCATCTGCTGCTG<br>CACCGGCTGCGGCAGCTGCTGCTTCAAGAAGTGCGGCAACTGCTGCGAC<br>GAGTACGGCGGCCACCAGGACAGCATCGTGATCCACAACATCAGCAGCC<br>ACGAGGAC
